# Accurate proteome-wide prediction of enzymes and catalytic sites using graph deep learning and protein language model

**DOI:** 10.1093/gigascience/giag056

**Published:** 2026-05-13

**Authors:** Yui-Lun Ng, Xiaomei Wang, Yingqi Li, Junzhe Huang, Hei Ming Lai, Jason Ying-Kuen Chan, Billy Wai-Lung Ng, Ho Ko, Ka-Wai Kwok

**Affiliations:** Department of Mechanical Engineering, Faculty of Engineering, The University of Hong Kong, Pok Fu Lam, Hong Kong; Department of Mechanical Engineering, Faculty of Engineering, The University of Hong Kong, Pok Fu Lam, Hong Kong; Department of Mechanical Engineering, Faculty of Engineering, The University of Hong Kong, Pok Fu Lam, Hong Kong; Division of Neurology, Department of Medicine and Therapeutics, Faculty of Medicine, The Chinese University of Hong Kong, Shatin, Hong Kong; Li Ka Shing Institute of Health Sciences, Faculty of Medicine, The Chinese University of Hong Kong, Shatin, Hong Kong; Margaret K. L. Cheung Research Centre for Management of Parkinsonism, Faculty of Medicine, The Chinese University of Hong Kong, Shatin, Hong Kong; Gerald Choa Neuroscience Institute, The Chinese University of Hong Kong, Shatin, Hong Kong; Division of Neurology, Department of Medicine and Therapeutics, Faculty of Medicine, The Chinese University of Hong Kong, Shatin, Hong Kong; Li Ka Shing Institute of Health Sciences, Faculty of Medicine, The Chinese University of Hong Kong, Shatin, Hong Kong; Margaret K. L. Cheung Research Centre for Management of Parkinsonism, Faculty of Medicine, The Chinese University of Hong Kong, Shatin, Hong Kong; Gerald Choa Neuroscience Institute, The Chinese University of Hong Kong, Shatin, Hong Kong; Department of Psychiatry, Faculty of Medicine, The Chinese University of Hong Kong, Shatin, Hong Kong; Department of Otorhinolaryngology, Head and Neck Surgery, Faculty of Medicine, The Chinese University of Hong Kong, Shatin, Hong Kong; Li Ka Shing Institute of Health Sciences, Faculty of Medicine, The Chinese University of Hong Kong, Shatin, Hong Kong; Gerald Choa Neuroscience Institute, The Chinese University of Hong Kong, Shatin, Hong Kong; Guangdong-Hong Kong-Macao Joint Laboratory for New Drug Screening, School of Pharmacy, Faculty of Medicine, The Chinese University of Hong Kong, Shatin, Hong Kong; Division of Neurology, Department of Medicine and Therapeutics, Faculty of Medicine, The Chinese University of Hong Kong, Shatin, Hong Kong; Li Ka Shing Institute of Health Sciences, Faculty of Medicine, The Chinese University of Hong Kong, Shatin, Hong Kong; Margaret K. L. Cheung Research Centre for Management of Parkinsonism, Faculty of Medicine, The Chinese University of Hong Kong, Shatin, Hong Kong; Gerald Choa Neuroscience Institute, The Chinese University of Hong Kong, Shatin, Hong Kong; Department of Psychiatry, Faculty of Medicine, The Chinese University of Hong Kong, Shatin, Hong Kong; Department of Mechanical Engineering, Faculty of Engineering, The University of Hong Kong, Pok Fu Lam, Hong Kong; Department of Mechanical and Automation Engineering, Faculty of Engineering, The Chinese University of Hong Kong, Shatin, Hong Kong

**Keywords:** enzyme function prediction, catalytic site prediction, protein language model, graph convolution network, deep learning

## Abstract

Identifying the enzyme functions of proteins and their catalytic residues is vital to our understanding of diverse cellular processes. However, existing frameworks that can concurrently determine the enzymatic functions and active sites of proteins are scarce, and still have much room for improvement in prediction performance. In this study, we present EC-LMGraph, a protein language model- and graph convolutional network-based framework to predict enzyme commission (EC) numbers from protein sequence features and structures, and saliency mapping to score representative residues attribute to the enzymatic functions. EC-LMGraph attained an average *F*_1_ score of 0.77 in 3rd-level EC number prediction, and 0.76 in 4th-level prediction, outperforming numerous other algorithms that were either sequence-based only, or additionally incorporated structural information. Benchmarking on the Mechanism and Catalytic Site Atlas dataset and a set of Parkinson’s disease-related proteins, we showed that EC-LMGraph showed a stronger emphasis on catalytic sites than the current state-of-the-art algorithm DeepFRI. Combining EC-LMGraph with AlphaFold2, our framework correctly determined the 3rd-level EC numbers of 229,160 proteins based purely on their predicted structures. We show that EC-LMGraph is capable of accurately predicting the 3rd/4th-level EC numbers, and pinpointing the key amino acid residues for many enzymes. EC-LMGraph is implemented and freely available at https://github.com/ngyuilun/EC-LMGraph.

## Introduction

Enzymes constitute a large class of proteins. Identifying enzymes and revealing how they function is crucial for understanding the mechanisms of cellular processes and disease pathophysiology. Known enzymatic functions, along with other protein characteristics (e.g., amino acid sequence, variants, and other molecular functions), are comprehensively documented in an open access database, the UniProt Knowledgebase [1] (UniProtKB), serving as an indispensable resource in biomedical research. With tremendous efforts over the past two decades, the number of sequence entries that had been manually annotated in UniProtKB reached 573,230 (as of 2025–04), which is, however, only around 0.23% of all recorded entries. The remaining vast majority of proteins (>252 million) could only be annotated using UniProt’s rule-based automatic annotation systems [2]. Even with high-throughput assays, identifying or verifying the enzyme-catalyzed reactions of the unreviewed proteins, or just annotating the known catalytic domains of enzymes, would still demand massive amounts of time and effort.

To accelerate the process of protein function determination, a method for accurate prediction of enzyme functions with robust functional site annotation is strongly desired. Most existing data-driven enzyme prediction frameworks are sequence-based [3–11] (i.e., relying on just the primary structure), as protein sequences are abundantly available. Although many of the sequence-based tools exhibit robust performances in inferring enzymatic functions, most of them focus solely on identifying the enzyme classes without indicating the putative catalytic residues. While some sequence-based methods may effectively identify catalytic sites that involve consecutive residues, due to the lack of geometric coordinates, they may not accurately detect catalytic or important residues that are physically close in the folded protein but distant in sequence (e.g., located in a different fold).

Structure-based methods have been commonly adopted in drug discovery (e.g., in virtual screening with molecular docking and molecular dynamics simulation) [12–14], since the interactions of proteins and other molecules are constrained by their shapes, surface charges, and other structural properties. Despite variations in the primary sequence, similar enzyme functions are often mediated by a small number of residues within the active domains with highly conserved local structures. The secondary and tertiary structures of proteins can therefore be promising predictors for their enzymatic functions [15–18]. To capture the intrinsic three-dimensional (3D) structures of proteins, a graph-based representation is a powerful approach, as the chemical properties of amino acids and their pairwise interactions can be represented by nodes and edges, respectively [19]. To utilize such a representation in machine learning frameworks, graph convolution operators can propagate node information, such that node properties can be aggregated and integrated by a graph convolutional network (GCN) [20].

In a recent work, DeepFRI [16] employed a long short-term-memory language model and GCN to annotate protein functions and detect functional regions with an average *F*_max_ score of ∼0.5–0.6. Nevertheless, the prediction performance still has room for improvement, particularly for the sequence feature extraction process that had not yet benefited from the recent advancements in large language models. Protein language models (pLMs), such as ProtT5 [21] and ESM-2 [22], leverage state-of-the-art transformer-based architectures [23] to generate embedded protein representations. These pLMs are comprised of billions of parameters (∼3 billion in ProtT5, and ∼15 billion in ESM-2), which enable them to extract relevant features from millions of sequences in UniRef (∼45 million on UniRef50, and ∼216 million on UniRef100). By utilizing these pre-trained models as a feature extraction module, it is possible to effectively capture the complex relationship between protein sequences and their functions. We hypothesized that the main limitation of several structure-based techniques for protein function prediction, specifically the relative scarcity of experimentally validated structures, could be alleviated by incorporating the protein sequence features captured by pLMs. These features have the potential to serve as supplementary input and enhance the performance in enzyme function prediction.

Here, we present EC-LMGraph, a deep learning framework that combines pLM with GCN to predict enzyme functions through learning graph representations of experimentally determined protein structures from the Protein Data Bank (PDB) [24]. In EC-LMGraph, we adopted a GCN architecture with a local extremum and graph convolution operators block, and performed training on three distinct, complementary types of data for each protein: the primary sequence, the pLM-embedded feature, and the structure graph. EC-LMGraph attained an average *F*_1_ score of 0.77 in 3rd-level enzyme commission (EC) number prediction, and 0.76 in 4th-level prediction, outperforming numerous other sequence-only or both sequence- and structure-based algorithms. To highlight protein regions essential for enzyme function prediction, we mapped the activation of EC-LMGraph on amino acid residues using model interpretability algorithms, and observed a high concordance between such regions and the catalytic residues curated in the Mechanism and Catalytic Site Atlas (M-CSA) database [25–27]. In head-to-head comparisons, EC-LMGraph outperformed DeepFRI on catalytic sites prediction for the M-CSA entries and a set of Parkinson’s disease (PD)-related enzymes. The EC-LMGraph source code and all prediction results are freely available and can be accessed online [28].

## Results

### Overview of the EC-LMGraph framework

**Figure 1:**
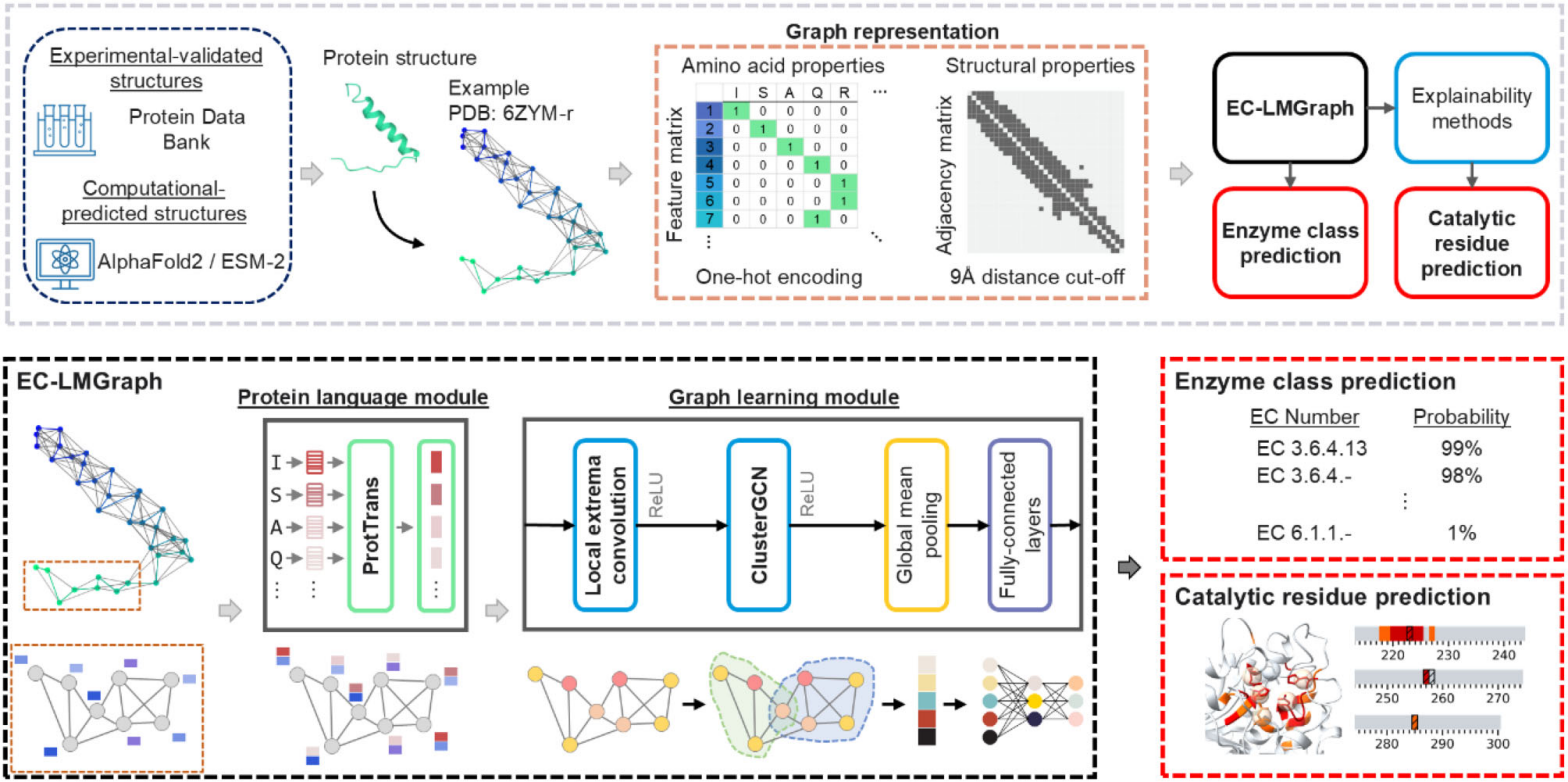
Overview of EC-LMGraph framework. Protein structure data, whether determined through experimental methods or predicted using computational approaches, can be taken as input in EC-LMGraph framework. The amino acid sequence and structure are represented as a feature matrix (with one-hot encoding) and an adjacency matrix (binarized with a 9 Å distance cut-off), respectively. The EC-LMGraph models employ a protein language model (ProtTrans) to generate feature embedding for each amino acid sequence, and a graph convolution module to learn and predict EC numbers from the input sequences, embedded features, and structure graphs. To identify which residue contributes to the prediction of the enzyme class, explainability methods were employed to calculate the importance of each amino acid residue. The importance values can be mapped to the corresponding amino acid residues such that a visual representation that highlights key residues based on their positions or spatial locations can be generated.

The goal of our framework is to train GCNs, which combine the sequence embeddings and graph representations of protein structures to predict their EC numbers and catalytic residues accurately (Fig. 1). Protein structure data, whether determined through experimental methods or predicted using computational approaches, can be taken as input in our framework. The structure of each protein was first pre-processed into an adjacency matrix and a feature matrix to obtain a protein graph format. The adjacency matrix encodes the pairwise Euclidean distances between the alpha carbon of amino acid residues, whereas the feature matrix encodes the amino acid identity. To prevent excess information transmission between graph nodes while preserving sufficient details of protein structures, we applied distance-thresholding to the adjacency matrix using 9 Å as the optimal cutoff (Fig. 1, Fig. S1). We analyzed the trade-offs between precision and recall across thresholds ranging from 6 Å to 13 Å (Fig. S1B). We observed that precision remained consistently high (>0.84) for cutoffs between 8 Å and 10 Å. Regarding recall, the model’s ability to retrieve true positives was superior when the cutoff was between 7 and 9 Å, with the 9 Å cutoff achieving the maximum recall of 0.72. Consequently, the 9 Å cutoff yielded the highest overall *F*_1_ score (0.78), effectively balancing the capture of relevant structural features against the inclusion of noise. To determine whether a threshold preference exists for specific enzyme classes, we further stratified the performance of 3rd- and 4th-digit predictions across major EC categories (Table S1). A distance cutoff of 9 Å consistently demonstrated robustness across diverse functional categories. At the 3rd-digit level, the 9 Å threshold is optimal for six of the seven classes; Hydrolases (EC 3) is the sole exception, slightly favoring a 10 Å cutoff. Regarding 4th-level predictions, the 9 Å cutoff remains the top performer for five classes, while Lyases (EC 4) and Isomerases (EC 5) exhibit minor preferences for 7 Å and 8 Å, respectively. Despite these slight variations, the 9 Å threshold provides the most consistent and accurate performance across the majority of enzyme functions. Hence, we empirically chose the 9 Å cutoff as optimal for defining residue contacts to effectively learn graph structures. Through these preprocessing steps, each protein structure was modeled as a node property-preserved, thresholded, and undirected graph.

EC-LMGraph employs a protein language module to transform the sequence into an efficient representation that captures the biological characteristics, and a graph convolution network module to disseminate the residual-level characteristics among residues located in close proximity in the three-dimensional space (Fig. 1). The protein language module incorporates a top-performing model from ProtTrans, namely ProtT5-XL-U50 [21]. The model was pre-trained using a vast dataset of over 2,122 M protein sequences from Big Fantastic Database (BFD) and further refined using additional 45 M protein sequences from the UniProt database. Note that this language model utilized self-supervised learning such that neither annotations nor labels were used to guide the training process, therefore it can make full use of the sequences in the UniProt database. The protein language module processes the input sequence and generates the corresponding feature embeddings. The adjacency matrix, feature matrix, and feature embeddings are then fed into the graph convolution module for learning the structure-function relationships (Fig. 1). The last layer of the graph convolution module is connected to global pooling operators and fully connected layers to output the final enzyme function predictions. In order to identify which residue contributes to the prediction of the enzyme class, we incorporated explainability methods to calculate the importance of each amino acid residue. By mapping the importance values onto the corresponding amino acid residues, our framework provides a visual representation that highlights key residues based on their positions or spatial locations.

We experimented on five types of graph convolutions, including the widely used graph convolutional layer (GCNConv) [20], graph attention (GATv2Conv) [29], hypergraph attention (HypergraphConv) [30], an efficient graph clustering algorithm (ClusterGCNConv) [31], and local extremum convolution (LEConv) [32], to investigate their efficacies in learning the structural representations of proteins. We compared the architectures incorporating these layers on (*i*) 3rd/4th-level EC class prediction performance, and (*ii*) number of catalytic sites identified for enzymes in the M-CSA database (Fig. S2A, also see later sections on catalytic site prediction evaluation). Overall, GCN attained the highest *F*_1_ score in EC number prediction (Fig. S2A), yet performed poorly on catalytic site prediction (Fig. S2B). ClusterGCNConv also demonstrated a relatively high score in EC prediction performance (Fig. S2A), and outperformed GCN for catalytic site identification (Fig. S2B). While LEConv allowed the most accurate catalytic site inference (Fig. S2B), an architecture solely based on LEConv exhibited more variable EC prediction performance (Fig. S2A). Balancing across the metrics, the combined LEConv-ClusterGCNConv (LE-ClusterGCN) architecture achieved relatively high scores in EC number classification (Fig. S2A), while permitting reasonably accurate catalytic site identification (Fig. S2B).

Many enzymes in nature are multi-functional, catalyzing multiple distinct reactions and thus carrying several EC numbers. To accommodate these cases, we have designed EC-LMGraph to compute independent probabilities for every EC class across the functional hierarchy (see the “Materials and methods” section). A challenge in multi-label classification is that the amount of training samples in each class is highly imbalanced, with many more negative than positive samples (e.g., for any given protein functional class, there are far more proteins that do not belong to the class) [33, 34]. Without measures to tackle this problem, graph neural network (GNN) classifiers often over-classify the majority of negative classes and fail to discriminate against the positive classes in the training processes [35]. Such an issue would be even more severe for 3rd/4th-level EC classes than 1st/2nd-level ones. To tackle the data imbalance issue and improve the classification performance, we employed a focal loss function [36] to guide the model to focus on learning from the minority class. Focal loss assigns a higher weight to the misclassified examples, thereby reducing the contribution of well-classified examples and increasing the contribution of misclassified examples during the training process. We evaluated the model’s performance using focusing parameters (γ) ranging from 1 to 4 and compared these results to the baseline binary cross-entropy (BCE) loss within a five-fold cross-validation setting. The model optimized with baseline BCE loss attained an *F*_1_ score of ∼0.68. By introducing the focal loss, we can accomplish a substantial improvement across all γ values tested, such that the *F*_1_ scores can consistently reach up to ∼0.77–0.78 (Fig. S3A). All tested γ values yield consistently high scores across folds, suggesting that the focal loss mechanism itself drives the performance gain. As the performance variations among the tested γ parameters were marginal, we adopted the standard setting of γ = 2. Apart from comparing different graph convolutions (Fig. S2), ablation studies were performed to assess the effectiveness of the protein language modules and graph network architecture. The use of the protein language module substantially improved the EC number prediction performance, from an average *F*_1_ score of ∼0.48 to ∼0.78 (Fig. S3B). To assess the importance of our proposed graph network architecture, additional models were trained by replacing the graph convolution module with fully connected network or 1D-convolutional neural network (Fig. S3B). Both architectures demonstrated a decrease in prediction accuracy, as the fully connected network combined with the protein language model only achieved an *F*_1_ score of 0.46, while the convolutional neural network attained a *F*_1_ score of 0.48. These results emphasize the importance of graph convolution module in learning and integrating structural information to achieve optimal performance. To assess the influence of mainstream protein language models on enzyme prediction, we trained LE-ClusterGCN architecture using embeddings from ProtT5-XL-U50 [21], ProtBert [37], and ESM-2 [22]. All three models demonstrated comparable performance, yielding robust *F*_1_ scores ranging from 0.77 to 0.78 (Fig. S3B). The overlapping confidence intervals (ProtT5: 0.76–0.79; ProtBert: 0.78–0.79; ESM-2: 0.78–0.78) indicate that the prediction accuracy is not heavily dependent on the specific pLM architecture. This consistency suggests that LE-ClusterGCN architecture effectively integrates structural graph features with high-dimensional sequence embeddings, regardless of whether the embeddings are derived from encoder-only (ProtBert, ESM-2) or encoder-decoder (ProtT5) architectures. Given this robustness, we chose ProtT5 embeddings as the representative sequence feature extractor for the subsequent analysis. Settling on the LE-ClusterGCN-based design, the parameters of EC-LMGraph were optimized through backpropagation of focal loss. The optimal model with the highest validation score was chosen for further evaluations.

### EC-LMGraph performance evaluation with temporal holdout validation

We composed a temporal holdout dataset to evaluate the performance of EC-LMGraph in a realistic scenario, by identifying newly annotated protein structures in the UniProtKB database between two releases, namely 2022_01 (Feb 2022) and 2025_02 (Apr 2025). Protein structures with enzyme functions annotated in release 2022_01 were used as the *training set*, while the *test set* contained those *newly annotated enzymes* in release 2025_02. Protein functions that were annotated in both releases were categorized as *previously known enzymes* and therefore were not included in the test set to avoid data leakage. This test set represents a diverse collection of protein functions that have been identified and documented over a one-year period. To focus on a more specific level of enzyme function (i.e., the 3rd/4th-digits), enzymes lacking annotations for their third digit were excluded. As the experimental validated structures deposited in PDB can represent the same protein sequence, a sequence clustering algorithm CD-HIT [38] was applied on the sequence of the PDB structures to cluster the data using an identity cut-off of 95%. This procedure reduced the presence of homologous proteins between the training set and test set. The training set contained 16,263 (∼83%) protein structures, and the test set contained 3,378 (∼17%) structures. To provide more reliable estimates of the model’s performance, we applied a five-fold cross-validation to the training set and employed iterative stratification [39] to ensure that the numbers of samples in each EC class were balanced. By employing a training process that includes five-fold cross-validation and utilizing the test set as an independent dataset, the models were evaluated on unseen protein structures such that any model overfitting can be detected and mitigated.

**Figure 2:**
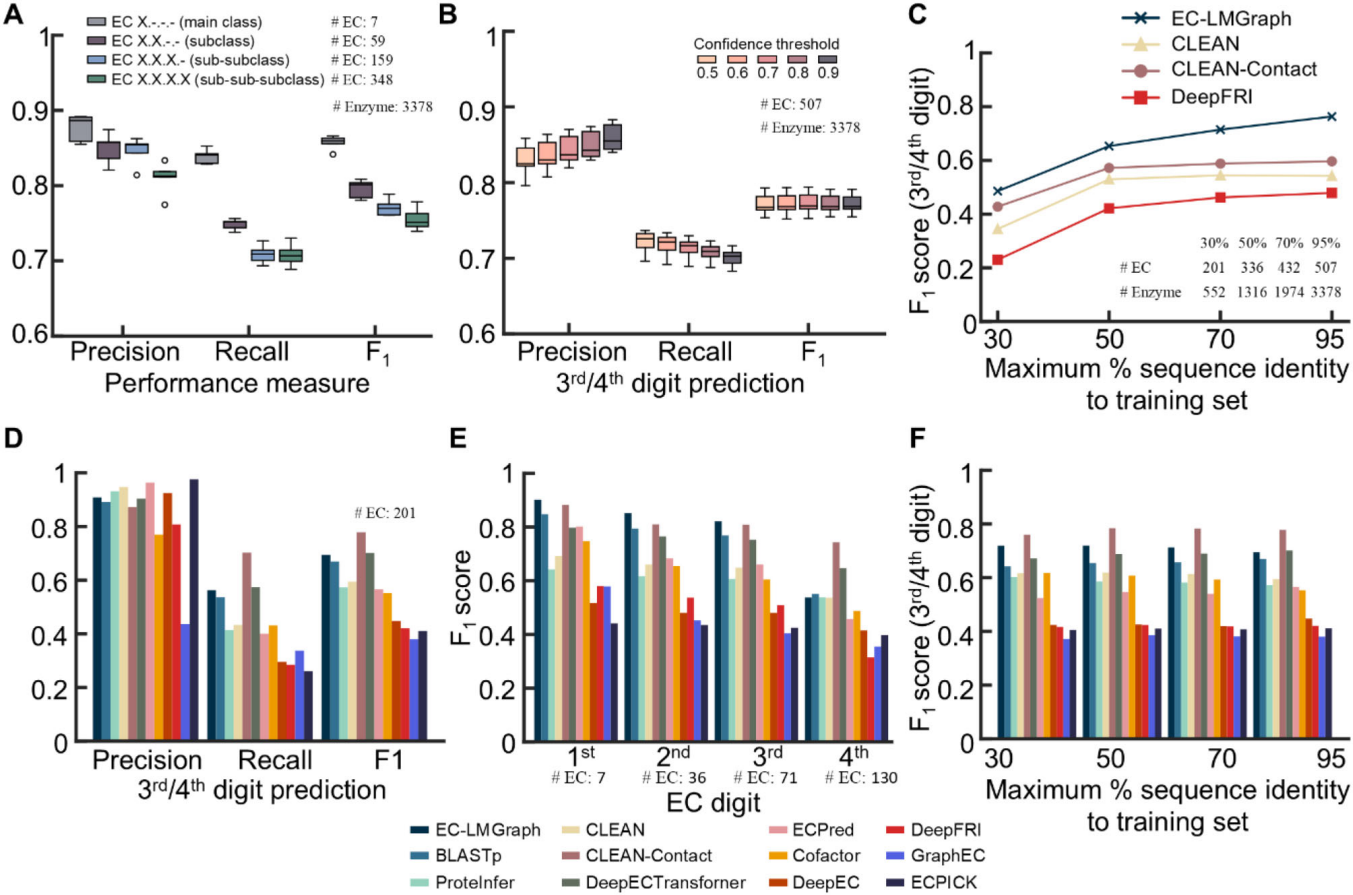
EC-LMGraph prediction performance. (A–C) Benchmarking results on newly annotated enzyme structures. (A) Performance of EC-LMGraph on the prediction of EC main class, subclass, sub-subclass, and sub-sub-subclass numbers, adopting a confidence threshold of 0.5. (B) Dependence of precision, recall, and *F*_1_ scores of EC-LMGraph on confidence thresholds ranging from 0.5 to 0.9. (C) Dependence of prediction performances on the training set maximum percentage of sequence identity cut-off for the compared methods. (D–F) Benchmarking results on newly annotated enzyme sequences. (D) and (E) Prediction performances on protein sequences with newly annotated EC numbers for the various compared methods. The input protein structures for the structure-based methods (i.e., EC-LMGraph, DeepFRI, and COFACTOR) were AlphaFold2-predicted structures. For (F), the dependence of prediction performances for 3rd/4th-digit EC numbers on the training set maximum percentage of sequence identity cut-off is shown for the compared methods.

We first trained and assessed the performance of our GCN architecture with six evaluation metrics, namely precision, recall, accuracy, specificity, *F*_1_ score, and Matthews Correlation Coefficient (MCC) (Fig. 2A, Fig. S3C). Given that this is a multi-label classification task with highly imbalanced classes, the evaluation metrics were computed using the micro-average, as it can reflect the overall performance and is less influenced by the performance of rare classes. When testing on the test set of 3,378 structures, the 3rd- and 4th-digit EC predictions of LE-ClusterGCN attained a minimum of 0.99 in accuracy and specificity (Fig. S3C), showing that the trained models could discriminate the negative class samples. On the performance of predicting positive cases, the models achieved mean recall scores of 0.71 for 3rd- and 4th-digit predictions (i.e., these models can predict the enzyme functions of at least 71% proteins in these classes) (Fig. 2A). Overall, we attained an average *F*_1_ score of 0.77 for 3rd- and 4th-level prediction (Fig. 2A, also see Fig. S3B for MCC). Regarding the accuracy of predicted positive cases, the GCN models demonstrated mean precision scores of 0.85 for 3rd-digit, 0.81 for 4th-digit, and 0.83 for 3rd/4th-digit predictions. To further validate the confidence of the predictions, higher thresholds for a positive prediction were applied to the predicted probabilities. Even with a threshold of 0.9, the *F*_1_ score attained remained at ∼0.77, while the precision can be improved to 0.86 at the expense of a slightly lower recall (Fig. 2B).

We benchmarked our models against the current state-of-the-art sequence-based and structure-based methods, namely CLEAN [4], CLEAN-Contact [5], and DeepFRI [16], respectively (Fig. 2C). We compared the results using various degrees of sequence similarity (see “Materials and methods” section), ranging from as high as 95% (3,378 structures) to down to 30% (552 structures). EC-LMGraph consistently demonstrated a higher predictive power than the other two methods, as evidenced by the higher *F*_1_ scores across all similarity cut-off values (Fig. 2C). Specifically, EC-LMGraph achieved *F*_1_ scores of 0.76 at 95% similarity cut-off, and 0.49 at 30% similarity cut-off, CLEAN-Contact achieved *F*_1_ scores ranging from 0.43 to 0.60, while CLEAN and DeepFRI achieved *F*_1_ scores of 0.34–0.54 and 0.23–0.48, respectively (Fig. 2C).

To broaden the applicability of EC-LMGraph to sequences without experimentally determined structures, we also assessed its performance against several sequence-based methods using computationally predicted structures. We obtained a list of 297 sequences from UniProtKB with newly identified EC functions (i.e., annotated between releases 2022_01 and 2025_02) and utilized AlphaFold2 to derive their corresponding predicted structures. We then compared the performance of EC-LMGraph against eleven other methods, including the sequence alignment method (BLASTp) [3], sequence-based deep learning methods (CLEAN [4], CLEAN-Contact [5], DeepECTransformer [6], ProteInfer [7], ECPred [8], DeepEC [9], GraphEC [10], and ECPICK [11]), and structure-based methods (DeepFRI [16] and COFACTOR [15]). In the overall combined 3rd/4th-level EC evaluation, CLEAN-Contact and DeepECTransformer showed strong performance with *F*_1_ scores of ∼0.78 and ∼0.70, respectively, while EC-LMGraph followed closely behind (∼0.69) (Fig. 2D). When breaking down the evaluation by independent hierarchical levels, EC-LMGraph consistently outperformed the other methods on broader functional classes, attaining the highest *F*_1_ scores for predicting the 1st-, 2nd-, and 3rd-digit EC classes (0.90, 0.85, and 0.82, respectively) (Fig. 2E). Up to the 4th-digit prediction, CLEAN-Contact achieved the highest *F*_1_ score (0.74), followed by DeepECTransformer (0.65). EC-LMGraph achieved results similar to those of BLASTp, CLEAN, and ProteInfer, with scores in the ∼0.53–0.55 range (Fig. 2E). The relative performances remained consistent across sequence similarity levels (Fig. 2F). These results suggest that utilizing precise 3D structural graphs gives EC-LMGraph a distinct advantage in capturing the core catalytic mechanisms that define the 1st-, 2nd-, and 3rd-digit EC classes. In contrast, sequence-based methods utilize massive databases to access a substantial volume of sequence examples, effectively capturing the fine-grained substrate specificities that characterize 4th-digit EC classes. An analysis of the EC-LMGraph training dataset reveals class imbalance as the functional hierarchy deepens (Table S2). While the 1st-digit level contains abundant data (median of 906 samples per class), the dataset would fragment severely owing to the deepen functional hierarchy. At the 4th-digit level, the median drops to just 8 training samples per class, with 67.7% of all classes containing ≤ 10 examples (Table S2). It is worth noting that by incorporating a protein language module pre-trained on large sequence databases, EC-LMGraph is able to learn through a substantially smaller annotated training set (i.e., limited to proteins with experimentally validated structures), even when the numbers of samples in the 4th-digit classes are especially small.

In addition to prediction accuracy, the computational speed of an enzyme function prediction framework is crucial for effectively analyzing large-scale proteome datasets. We conducted an analysis to evaluate the computation time of these EC number prediction frameworks by randomly selecting 100, 200, 500, and 1,000 proteins and recording the time taken (Fig. S3D). Among the twelve evaluated methods, EC-LMGraph ranked fifth (∼240 seconds for 1,000 protein structures) and outperformed other structure-based methods such as DeepFRI and COFACTOR. Despite the substantial number of parameters (∼3B) in the protein language model, the architecture of EC-LMGraph allows for effective handling of the embedded protein representations and structural information while maintaining a comparable computation speed to other sequence-based methods.

These findings thus highlighted the advantages of combining protein language models with structure-based approach in predicting enzymatic functions, especially when the protein structure is available or can be predicted using computational methods.

### Catalytic sites prediction based on EC-LMGraph saliency mapping

Proteins possess their enzymatic functions in specific regions where substrates bind to the catalytic domain(s) and undergo chemical reactions. Apart from determining the reaction(s) catalyzed, pinpointing the key amino acid residues constituting the catalytic sites is crucial to understanding the mechanisms of catalysis. Identification of catalytic sites is a complicated process, as these usually occupy only less than 1% of the volume of an enzyme [26], while mutagenesis experiments are time-consuming and resource-intensive. To explore the relationship between learned graph features and catalytic sites, we mapped the activation values of the EC-LMGraph models onto amino acid residues using various explainability methods, including saliency map (Saliency) [40], multiplies gradient with respect to input (InputXGradient) [41], guided backpropagation (GuidedBackprop) [42], and deconvolution (Deconvolution) [43]. These explainability methods were selected based on their model-agnostic nature, as they can be applied to any type of graph layer, regardless of its specific characteristics or structure. This characteristic enabled the selected explainability methods to seamlessly handle the *five* different types of graph layers that were tested (Fig. S2B), without encountering any limitations. The Saliency method was chosen as the optimal explainability method given that it demonstrated the highest correlation between the predicted and annotated catalytic sites (Fig. S4). The Saliency map derives the node importance based on the magnitude of gradient. A high gradient value suggests that this input node could lead to significant impact on the model’s output, thus highlighting the importance of that residue. By highlighting important amino acid residues for correct classification, we hypothesized that the so-obtained localization map can predict a subset of catalytic sites.

**Figure 3:**
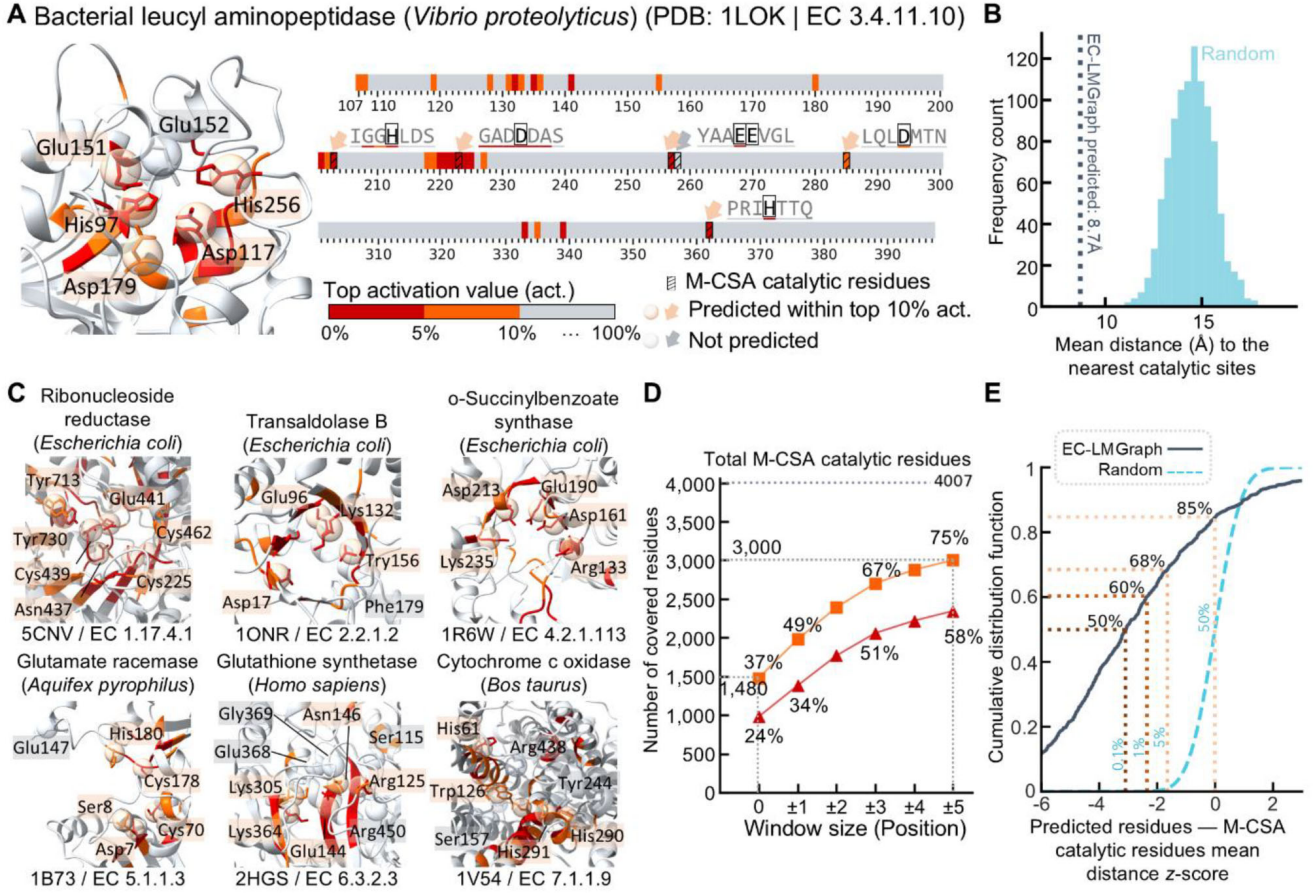
EC-LMGraph saliency mapping and catalytic residue prediction. (A) EC-LMGraph–Saliency-predicted catalytic residues of a bacterial leucyl aminopeptidase (PDB: 1LOK). Residues are colored according to the EC-LMGraph saliency values. The ground truth catalytic sites annotated in the Mechanism and Catalytic Site Atlas (M-CSA) are marked above the amino acid chain for reference. (B) Comparison of the mean distance (Å) between residues with top 10% activation values and the nearest M-CSA-annotated catalytic sites vs. that expected by random. The mean distance distribution expected by random was generated by randomly sampling the same number of residues across the whole amino acid chain. (C) Examples of enzymes from various species that belong to the different EC classes, showing residues with top EC-LMGraph saliency values and those that coincide with M-CSA-annotated catalytic sites. (D) Total number of M-CSA catalytic residues that are covered by residues with top 5% or 10% EC-LMGraph saliency values. (E) Cumulative distribution plot of the mean distance *z*-scores of the EC-LMGraph–Saliency predicted residues (to the nearest catalytic residues), compared to that expected with randomly drawn residues.

We evaluated the performance of EC-LMGraph catalytic site prediction using M-CSA [25–27], an expert-annotated database documenting enzyme catalytic residues derived from experiments. A catalytic residue was considered to be predicted by EC-LMGraph if (*i*) the 3rd-/4th-level EC number prediction for the protein structure is correct, and (*ii*) the activation value at the residue is among top 10% across the whole amino acid chain. Since the exact number of catalytic sites in an uncharacterized enzyme is rarely known, establishing the top 10% of predicted residues as the putative active site could help prioritize the catalytic hotspots for ease of downstream experimental validation. This approach ensures standardized performance comparisons by evaluating an algorithm’s capacity to rank actual catalytic residues within its top predictions. Furthermore, this relative threshold does not introduce length-dependent bias, as the random probability of capturing true sites remains statistically constant regardless of enzyme length. From M-CSA, we identified 4,007 catalytic residues for the protein structures with correct sub-subclass predictions in our dataset. As an illustrating example, for the bacterial leucyl aminopeptidase from *V. proteolyticus* (PDB: 1LOK) (EC 3.4.11.10), 5 out of 6 catalytic residues were predicted by EC-LMGraph–Saliency (Fig. 3A), and this was extremely unlikely to occur by chance (Fig. 3B). Likewise, further examples from the other major EC classes, including *E. coli* ribonucleoside reductase (PDB: 5CNV; EC 1.17.4.1), transaldolase B (PDB: 1ONR; EC 2.2.1.2), o-succinylbenzoate synthase (PDB: 1R6W; EC 4.2.1.113), *A. pyrophilus* glutamate racemase (PDB: 1B73; EC 5.1.1.3), human glutathione synthetase (PDB: 2HGS; EC 6.3.2.3), and rat cytochrome c oxidase (PDB: 1V54; EC 7.1.1.9), as depicted in Fig. 3C, highlighted EC-LMGraph–Saliency’s capability in predicting catalytic sites for enzymes of all seven major EC classes across different species.

Overall, 1,480 M-CSA-annotated residues were located at sites with top 10% EC-LMGraph saliency values, accounting for 37% of the total catalytic residues on proteins with correct EC 3rd/4th-digit predictions (Fig. 3D). Since residues near the catalytic ones could also be important for determining the local conformation and hence enzyme function, we postulated that EC-LMGraph activation sites may cluster nearby (as also illustrated by the examples shown in Fig. 3A, C). Consistently, the distances between EC-LMGraph–Saliency-predicted residues were much closer to the M-CSA catalytic sites than by chance (Fig. 3E), with 75% of the annotated catalytic residues locating within ±5-residue windows of top 10% EC-LMGraph activation sites (Fig. 3D).

**Figure 4:**
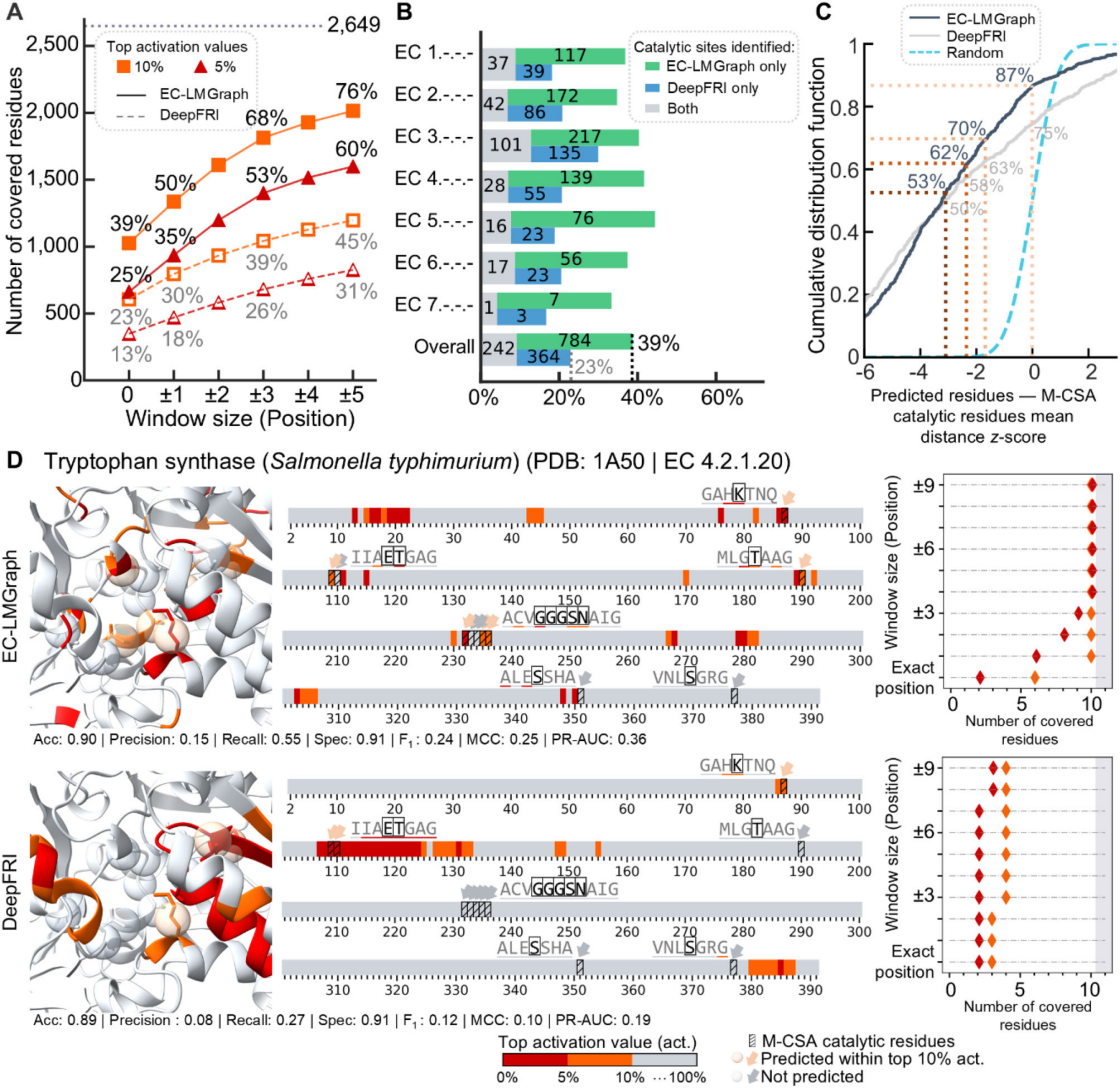
Benchmarking EC-LMGraph against DeepFRI. (A) Total number of catalytic residues annotated in the Mechanism and Catalytic Site Atlas (M-CSA) that are covered by residues with top 5% or 10% activation values, with EC-LMGraph or DeepFRI. (B) Numbers of M-CSA catalytic sites identified by each method, overall and for the enzymes of each main class. (C) Cumulative distribution plots of the mean distance *z*-scores of the EC-LMGraph–Saliency and DeepFRI–Grad-CAM predicted residues (to the nearest catalytic residues), compared to that expected with randomly drawn residues. (D) Prediction of catalytic residues for the *Salmonella typhimurium* tryptophan synthase (PDB: 1A50, chain B) by EC-LMGraph (upper panels) vs. DeepFRI (lower panels).

We additionally benchmarked the performance of EC-LMGraph against DeepFRI on the M-CSA dataset, specifically analyzing cases for which both models produced positive predictions. Higher percentages of M-CSA-annotated residues were located at sites with top 5% or 10% activation values for EC-LMGraph than DeepFRI (Fig. 4A), and similarly when we considered the proportions within ±5-residue windows (Fig. 4A). When considering the utilization of the 5% or 10% activation values as predicted sites and observing the ratio of correctly predicted sites over the overall predicted sites (Fig. S5A), the percentage of correctly predicted sites by EC-LMGraph is consistently higher than DeepFRI. The results indicate that the predicted sites by EC-LMGraph have a higher likelihood of being actual catalytic sites compared to DeepFRI. In addition to evaluating the prediction performance of catalytic residues based on amino acid position, we also tested using window sizes defined by a sphere radius ranging from 3 Å to 7 Å (Fig. S5B). A window size of 0–3 Å did not include any additional residues in the neighborhood; hence, the result was the same as using a residue position-based window size of 0 (i.e., exact position). We also observed that the position window sizes of ±1, ±3 and ±5 demonstrated similar prediction performance compared to Euclidean distance window sizes of 4 Å, 6 Å, and 7 Å, respectively. These suggested that the prediction accuracy achieved based on amino acid position aligns closely with the performance achieved using window size based on Euclidean distance. Classifying the M-CSA entries by the EC main class numbers, the superior catalytic site prediction performance of EC-LMGraph generalized across enzymes from the sub-subclasses of all seven main classes (Fig. 4B). In line with these, we found shorter distances between EC-LMGraph activation sites and the M-CSA annotated sites, than that obtained with DeepFRI (Fig. 4C, also see Fig. 4D for illustrating examples). EC-LMGraph also consistently outperformed DeepFRI across all evaluation metrics (Table S3). Notably, it achieved substantially higher recall (0.390 vs. 0.224) for superior coverage, more than doubled the MCC score (0.109 vs. 0.047), and improved the area under the precision-recall curve by ∼65% (0.220 vs. 0.133).

Collectively, these results showed that the features EC-LMGraph learned for enzyme function prediction tend to reside at or near catalytic residues, with EC-LMGraph being more catalytic site-emphasized than DeepFRI.

### Proteome-wide enzyme class and functional residue prediction based on predicted structures

Although advancements in X-ray crystallography and cryogenic electron microscopy have greatly accelerated structure identification (∼11,000 entries per year [44]), the growth of the protein structure dataset is still far from keeping pace with new sequence discovery. A common bottleneck for all structure-based function prediction frameworks is therefore the relative scarcity of experimentally determined protein structures. Building on the observation that applying EM-LMGraph on predicted structures performed favorably in comparison to sequence-based methods (Fig. 2D–F), we speculated that the use of high-quality predicted structures such as those by AlphaFold [45, 46] and RoseTTaFold [47, 48] would allow a much broader applicability of EC-LMGraph.

**Figure 5:**
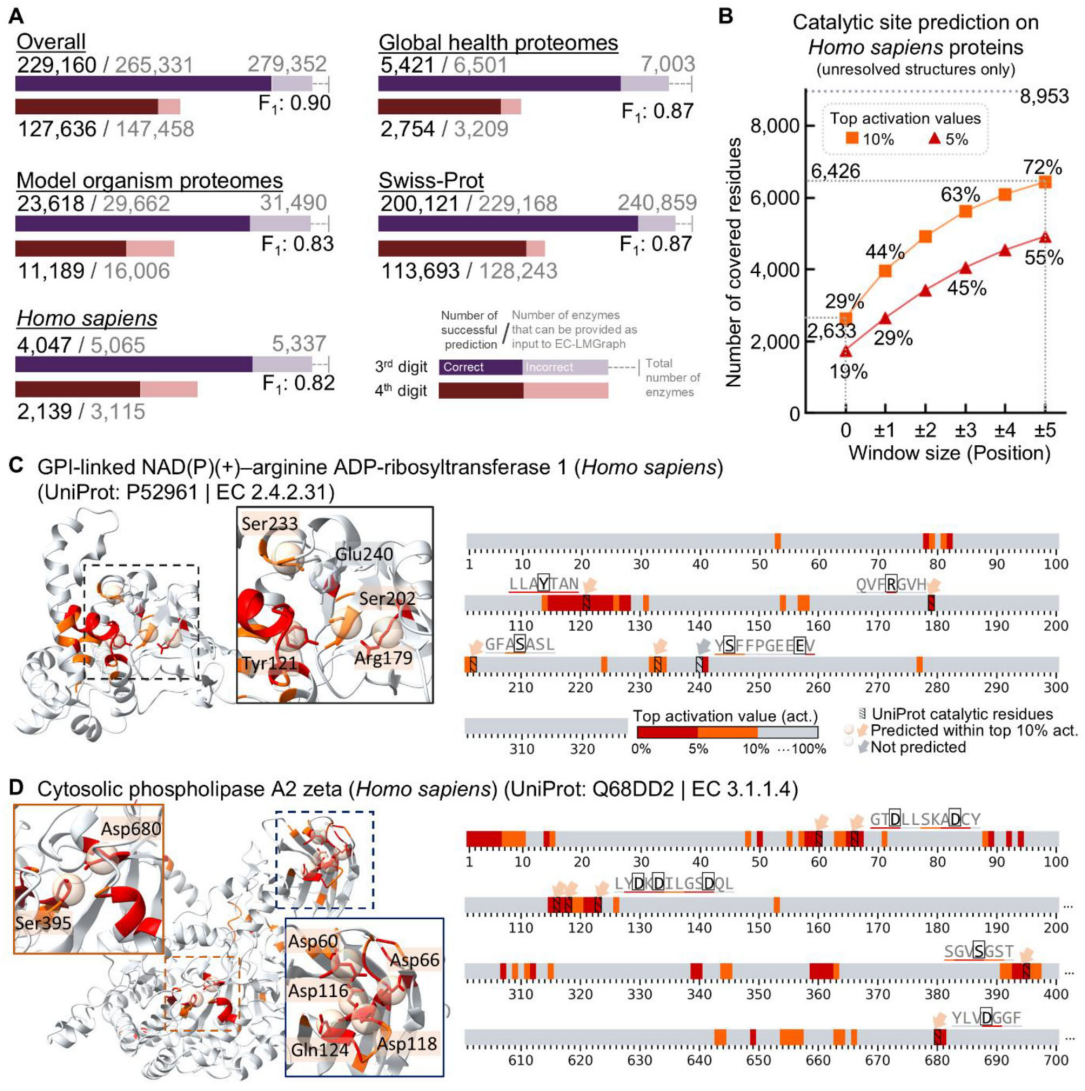
Enzymatic prediction based on AlphaFold2 (AF2)-predicted full-length structures. (A) Number of enzymes with EC numbers correctly predicted by EC-LMGraph based on AF2-predicted structures. (B) Numbers and proportions of catalytic residues covered by residues with top 5% or 10% EC-LMGraph saliency values based on the annotation of *Homo sapiens* proteins from UniProt. (C) and (D) EC-LMGraph–Saliency-predicted residues on AF2-predicted structures.

We retrieved the fourth release of 995,411 AlphaFold2-predicted structures, including model organism proteomes (326,175), global health proteomes (238,274), and Swiss-Prot (430,962). Among these, we identified 279,352 protein structures with enzyme functions annotated in UniProtKB (excluding unreviewed entries from UniProtKB TrEMBL), among which 265,331 came from the EC sub-subclasses for which we had sufficient dataset sizes for GCN training. EC-LMGraph correctly determined the 3rd-level EC numbers for 229,160 (∼86.4%) of the proteins based on predicted structures (Fig. 5A). Among entries with 4th-level EC numbers annotated, our method accurately predicted 127,636 out of 147,458 proteins, representing 86.6% of the annotated records. For the human proteome, EC-LMGraph correctly predicted 4,047 out of 5,065 (79.9%) 3rd-level, and 2,139 out of 3,115 (68.7%) 4th-level EC numbers (Fig. 5B). We also quantified the prediction performance with evaluation metrics (pooled across sub-subclasses, see Table S4). Across species, the *F*_1_ scores and MCCs reflected the highest model performances for human and several model organisms, including mouse, rat, zebrafish, and fruit fly, with *F*_1_ scores and MCCs of 0.72–0.78 (Table S4). EC-LMGraph therefore permits proteome-wide prediction of enzyme functions in various species even with only algorithm-predicted structures.

As EC-LMGraph relies on the geometric arrangement of residues, the reliability of the AlphaFold2-predicted structure is crucial. We utilized the pLDDT (predicted Local Distance Difference Test), a per-residue confidence metric from AlphaFold2, to assess the confidence of the predicted atomic positions. To ensure the rigor of our predictions on computationally generated structures, we investigated the impact of these confidence scores on model performance by categorizing proteins into three groups: High (pLDDT > 90), Medium (70 < pLDDT ≤ 90), and Low (pLDDT ≤ 70). We observed that the quality of the predicted structure correlates with the accuracy of EC number prediction (Fig. S6). For the 3rd/4th-digit predictions, EC-LMGraph achieve an *F*_1_ scores of 0.921 (95% CI: 0.920–0.922) for high-confidence structures and 0.817 (95% CI: 0.814–0.820) for medium-confidence structures. Performance in the low-confidence group decreased to 0.615 (95% CI: 0.602–0.627). These results demonstrate that EC-LMGraph is highly effective for structures with high pLDDT scores, whereas predictions derived from low-confidence models (pLDDT ≤ 70) should be interpreted with caution.

We further tested whether the saliency mapping of EC-LMGraph can be similarly adopted for the identification of important functional residues based on predicted structures. We examined this on human proteins, by selecting a set of previously unsolved sequences and compared the top 10% saliency values with the UniProt-annotated catalytic residues. For proteins with correct EC 3rd/4th-digit predictions, 2,633 (∼29%) UniProt-annotated residues were located at sites with top 10% EC-LMGraph saliency values. As illustrating examples, for the human GPI-linked NAD(P)(+)–arginine ADP-ribosyltransferase 1 (UniProt: P52961; EC 2.4.2.31) and cytosolic phospholipase A2 zeta (UniProt: Q68DD2; EC 3.1.1.4), sites with top EC-LMGraph saliency values correspond very well with UniProt-annotated catalytic residues (Fig. 5C, D). Collectively, based on these results, we concluded that a combined AlphaFold2–EC-LMGraph–Saliency approach can be used to generate hypotheses regarding the positions of functionally important amino acid residues for proteins even with only predicted structures, highlighting putative catalytic or functionally important sites. In principle, EC-LMGraph can also be used in combination with other protein structure prediction algorithms.

### Enzymatic prediction for Parkinson’s disease-related proteins

**Figure 6:**
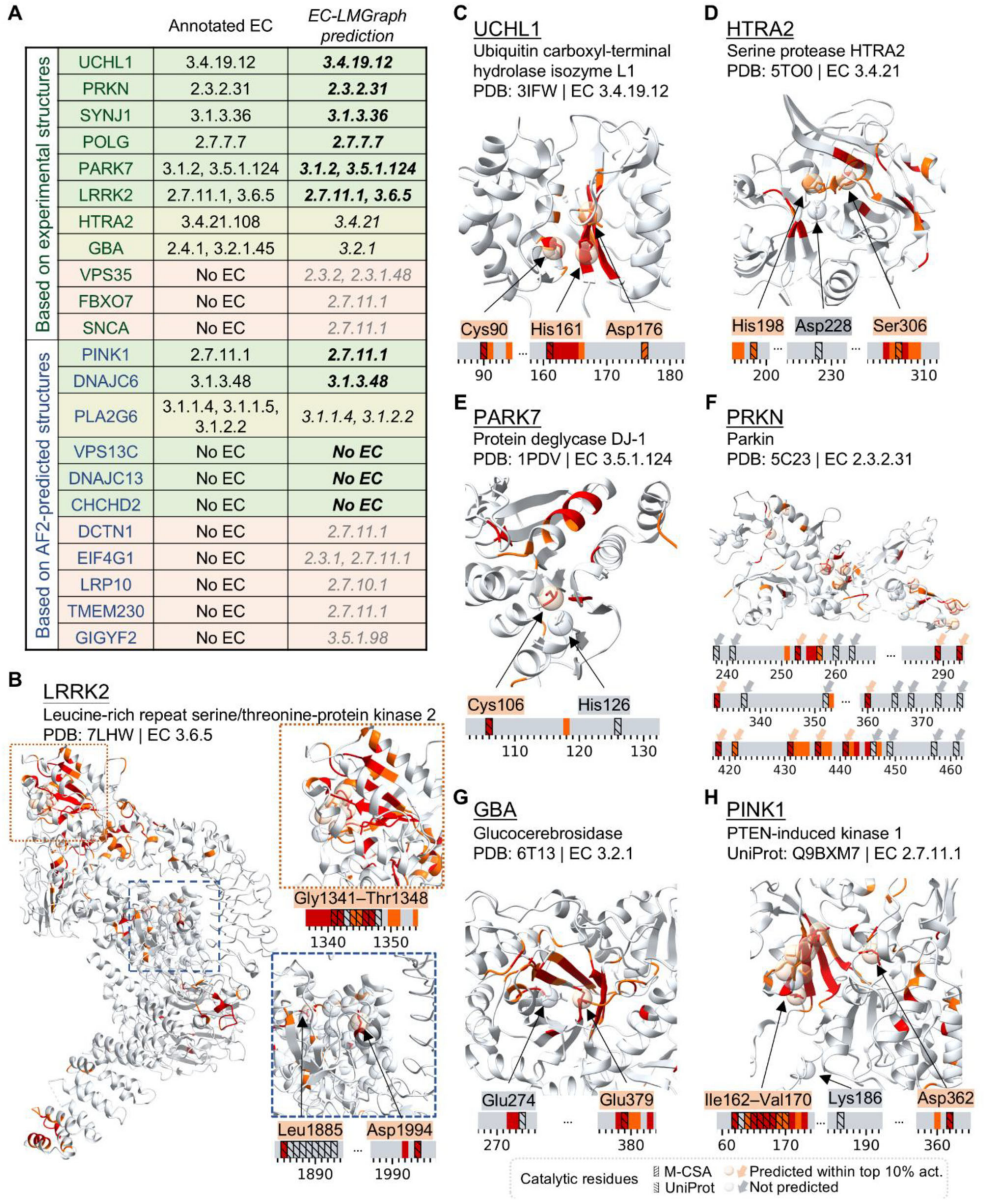
Enzymatic prediction for Parkinson’s disease (PD)-related proteins. (A) EC-LMGraph predictions for a set of PD-related proteins. (B–H) Catalytic sites prediction for PD-related enzymes using EC-LMGraph.

To evaluate the capability of EC-LMGraph in identifying disease-related enzyme functions, we performed a case study on a set of known human PD-related proteins [49–51]. The structures of these proteins had been excluded from the training set, and we tested whether EC-LMGraph could correctly predict their enzyme functions (Fig. 6A). In total, EC-LMGraph gave correct predictions for 8 (6 for 4th-digits) out of the 11 PD-related proteins examined with experimentally determined structures. These include the GTPase domain and its catalytic sites on leucine-rich repeat serine/threonine-protein kinase 2 (LRRK2, Fig. 6B), ubiquitin carboxyl-terminal hydrolase isozyme L1 (UCHL1, Fig. 6C), serine protease HTRA2 (Fig. 6D), protein deglycase DJ-1 (Fig. 6E), parkin (Fig. 6F), glucocerebrosidase (GBA, Fig. 6G), as well as SYNJ1 and POLG (Fig. 6A). For the other PD-related proteins with no known enzyme functions, EC-LMGraph incorrectly predicted VPS35 with EC 2.3.2 and 2.3.1.48, while FBXO7 and SCNA were mis-classified as EC 2.7.11.1.

Similar results were obtained using AlphaFold2-predicted structures, where EC-LMGraph gave consistent results for UCHL1, HTRA2, parkin and GBA, and additionally predicted the known enzyme sub-subclass number for PTEN-induced kinase 1 (PINK1, Fig. 6H) and DNAJC6 (Fig. 6A). While DeepFRI also predicted the enzyme sub-subclass numbers for UCHL1, HTRA2, DJ-1, and parkin, EC-LMGraph consistently showed active site predictions closer to the known experimentally identified catalytic sites annotated in M-CSA and/or UniProt than DeepFRI (see Fig. S7). We thus concluded that EC-LMGraph will complement existing algorithms, and can be applied to predicting protein enzyme functions and corresponding active sites in disease-related settings.

## Discussion

Recent advancements in machine learning methods have allowed unprecedented predictions of protein structure [46, 47], function [4, 7–9, 15–17, 52–54], protein–protein interaction [55–58], protein-substrate binding site [59, 60], and protein–nuclei acid interaction [61–63]. With EC-LMGraph, we made further advancements in protein enzyme function prediction. Combining (*i*) a protein language module that has learned an efficient representation of protein sequences, (*ii*) an architecture incorporating local extrema and graph clustering convolution operators blocks, (*iii*) the use of protein feature matrix and protein structure graphs with empirically optimized distance thresholding, and (*iv*) the increasing availability of solved enzyme structures, EC-LMGraph is capable of accurately predicting the 3rd/4th-level EC (i.e., enzyme sub-subclass or sub-sub-subclass) numbers, and pinpointing the key amino acid residues that constitute the catalytic sites for many enzymes.

Each component of EC-LMGraph is crucial to its performance. For instance, the incorporation of the protein language module led to substantial improvement in prediction accuracy compared to utilizing the graph convolution module alone. This substantiates the role of the protein language module as an effective sequence feature extractor, providing a more comprehensive representation of sequence data than conventional one-hot encoding or LSTM-based models [21]. For the graph convolution modules, the integration of the LEConv layer in the network can significantly boost the performance of catalytic site identification. This convolutional layer facilitates the consideration of both local and global node importance within the protein graph; thereby, the resultant activation mapping can be highly correlated to the annotated catalytic site [32]. In addition, the ClusterGCNConv utilizes a graph clustering algorithm to identify the most crucial nodes within a subgraph, thereby limiting the neighborhood search to this subset. Such an approach enables efficient learning of protein structural information, even for large proteins (e.g., those with over 1,000 amino acids) [31].

The learned graph features in EC-LMGraph can be interpreted as relying on the key residues in protein chains which are more conserved in each enzyme class or sub-subclass. In stringent head-to-head benchmarking, we showed that EC-LMGraph is a more catalytic site-emphasized than DeepFRI. As mutagenesis experiments are relatively costly and time-consuming, EC-LMGraph serves as a constructive tool to streamline experimental design by prioritizing the choice of residues from full sequences to those among or in the vicinity of top ∼10% activation scores. This may especially benefit studies demanding for identifying mutations in enzymes, accounting for changes of function and disease phenotypes [64]. In future work, it would be valuable to extend the framework to predict the mutation-induced loss of catalytic functions, due to the increasing need to understand the impact of genetic variations on enzyme activity. Experimental determination of mutated protein structures is often limited, making it challenging to directly assess the functional consequences of mutations. Thus, accurate prediction of protein structures for mutated variants would be a crucial aspect in training a framework capable of predicting the loss of catalytic function from these mutations. It is important to note that the negative labels in the training data should indicate a complete loss of function. Cases where mutations result in reduced metabolic rates instead of a complete loss may require specific handling or labeling strategies. By addressing these considerations, the framework can be extended to handle mutated protein structures and accurately estimate the impact of mutations on catalytic functions.

The prediction performance of EC-LMGraph certainly still has room for further improvement. With the number of solved enzyme structures rapidly increasing year by year, the capability of EC-LMGraph will also continue to grow, especially for sub-subclass models, which had few samples from prior to our temporal holdout dataset cut-off date to be trained on. Further architectural variations can be explored. For example, additional details in protein structure representations may be incorporated. Apart from using a contact map to represent whether the residues are within the predetermined thresholds, the inter-residue distance, orientation angles between adjacent residues or sidechain dihedral angles could be added to provide a more complete description of local protein conformations. EC-LMGraph currently utilizes static structural representations that do not explicitly account for environmental conditions like pH or temperature. While an enzyme’s intrinsic 3D fold dictates its fundamental EC classification, environmental factors largely influence its reaction kinetics and conformational dynamics. Incorporating environmental metadata is currently bottlenecked by data scarcity, future integration of high-throughput assays or molecular dynamics simulations could potentially overcome these hurdles. For many enzymes, difference in one amino acid residue with crucial physicochemical properties is sufficient to render a given catalytic site inactive. For algorithm-identified catalytic residues, additional filtering based on the known requirements for a given reaction could be used to refine prediction results. In addition to function predictions, recent deep learning approaches have also advanced in predicting enzymatic kinetic parameters. Methodologies utilizing protein language models and graph neural networks, such as UniKP [65] and DEKP [66], have been successfully applied to estimate turnover numbers and Michaelis constants directly from protein sequences and substrate structures. While these tools focus on the quantitative mechanics of enzyme-substrate efficiency, EC-LMGraph could complement this broader ecosystem by focusing on the preceding step: accurate, structure-aware prediction of the enzyme function and the localization of its catalytic sites. In future works, incorporating these with other variations in the design of GCNs may enable even more superior enzymatic function and active site predictions.

## Materials and methods

### EC number annotations of protein chain

Experimental validated structures from the PDB [24] and their corresponding EC number annotations from the UniProtKB [1] were retrieved to train the models. We first had to analyze the annotations in UniProtKB to compile a comprehensive catalog of protein functions for the PDB entries. This process is necessary because many of the structures deposited in the PDB can be multimers of several identical subunits (e.g., 1JU6: a homodimer structure consisting of two identical units) or structural subunits of large proteins (e.g., 7BV1: a complex consisting of the NSP7, NSP8, and NSP12 parts of R1AB_SARS2, where only NSP12 is found to have enzymatic functions). Therefore, the entire structure had to be separated into individual peptide chain structures to ensure that the EC number annotations were accurately associated with the corresponding chain structure.

Given the enzyme function annotations provided by UniProtKB accessions, protein chains were first extracted as sequence intervals labeled with EC numbers. Using the cross-references in each accession, a list of PDB identifiers associated with the sequence intervals was then collected, together with their chain and sequence positions. The start and end positions of the peptide chain were mapped onto the sequence intervals, given with the length-overlap and fraction-overlap computed. To avoid small ligands or protein connector subunits inaccurately categorized as enzymes, mapped records were excluded if (*i*) the length-overlap was <100 residues, and (*ii*) the fraction-overlap was <90%. Short sequences (i.e., <100 residues) therefore needed to have a high fraction-overlap of over 90% to be included. Records with annotated EC number only up to the 2nd-digit were excluded (i.e., only those with at least third-level EC numbers were included).

### Dataset construction

*Training set*: A temporal holdout validation setting was employed to evaluate the performance of EC-LMGraph in a realistic scenario. The records were categorized into training or test data based on the difference in EC annotation between successive UniProtKB releases. Specifically, protein structures with EC functions annotated in release 2022_01 were categorized as the training set. With the above EC number annotations steps, 12,355 sequence intervals and their EC annotations were obtained from release 2022_01 (dated 2022 Feb 23), along with the corresponding 85,879 structures from the PDB database. Owing to the presence of numerous duplicate structures within the PDB, it is necessary to employ a sequence clustering algorithm to group the PDB structural data based on their sequence similarity. CD-HIT [38] algorithm was chosen, and an identity cutoffs of 95% was applied to minimize redundancy in the training set, resulting in a reduction of the training set to 16,263 protein structures.

*Newly annotated enzyme structure set*: To identify a set of newly annotated enzymes, we compared the annotation difference between UniProt release 2022_01 and a later release 2025_02 (dated 2025 Apr 9). Entries with a new EC number emerged in release 2025_02 were categorized as newly annotated enzymes. To ensure redundancy removal between training set and test set, the CD-HIT clustering algorithm with an identity cutoff of 95% was applied to these newly annotated records and the training set. After removing redundancies, records with a structural model deposited in PDB were categorized as newly annotated enzyme structures, which consist of 3,378 unique structures. Similarly, lower degrees of sequence identity cutoffs (30%, 50%, 70%) were applied to reduce sequence redundancy and demonstrate model robustness.

*Newly annotated enzyme sequence set*: Following the above steps, records without any structural model deposited in PDB were categorized as newly annotated enzyme sequences. Records annotated using the UniProt rule-based system were also excluded to ensure this set of newly annotated enzyme sequences was based on the latest experimental evidence. Several enzyme classes could have identified sequences but lack known protein structures, these classes were not included to ensure a fair comparison between sequence-based and structure-based methods. After exclusion, this newly annotated enzyme sequence set consisted of 297 sequence records, and their corresponding AlphaFold2-predicted structures were retrieved. Lower degrees of sequence identity cutoffs (30%, 50%, 70%) were applied to assess performance and evaluate robustness.

*M-CSA set*: M-CSA [25–27] serves as a comprehensive database specifically documenting enzyme catalytic residues derived from experiments. A total of 991 PDB entries and their corresponding 5,038 catalytic site residues were retrieved. To remove high similarity side chains or structures, CD-HIT with an identity cutoff of 95% was applied to cluster a non-redundancy set. This reduced the M-CSA set to 937 unique protein chains and 4,469 catalytic site residues.

*AlphaFold2-predicted structure set*: The latest release (fourth release) of AlphaFold2 Database [45, 46] contains over 200 million computational predicted protein structures. Not only does it provide the predicted structures of human proteome and the proteomes of 47 other key organisms, but it also covers the manually curated set from UniProtKB Swiss-Prot. Among these, we collected 995,411 predicted structures, including 326,175 model organism proteomes, 238,274 global health proteomes, and 430,962 Swiss-Prot. After excluding unreviewed entries from UniProtKB TrEMBL, this set consisted of 657,773 computationally predicted structures.

### Protein graph formation

Protein graph data structure can be formed, with (*i*) the protein constituents (i.e., atoms and residues) represented by graph nodes, and (*ii*) metrics calculated from the intrinsic 3D atomic coordinates as the graph edges. For our graph models, the nodes are defined at the amino acid residue level. The residue coordinates were primarily based on the atomic coordinates of alpha carbon, while beta carbon and the centroid of each amino acid will be taken as reference in the absence of alpha carbon. This mitigates the effect of any missing backbone carbon atoms due to the low resolution of some experimental structures. To transform the twenty standard amino acid residues using the one-hot encoding [67] method, a node feature (sparse) matrix, ${{\bf X}} \in {\{ {0,1} \}}^{L \times 20}$ is formed, where each matrix column denotes one amino acid type and *L* is the number of residues in the protein sequence.

To describe the spatial relationship between amino acid residues, we use the inter-residue separation of residues (in Å) to construct a contact map for each protein. A distance cutoff value was adopted to define which edges should be kept. This cutoff was empirically optimized to preserve the structural representation of the catalytic functional regions while discarding unnecessary edges, thereby avoiding noise propagation to neighbors and reducing the computational times required for model training. In prior works [16, 68], cutoff values of 6 Å or 10 Å were usually chosen (i.e., distances smaller than which define in-contact residue pairs) for protein structure graphs. As a result, a contact map of a given protein was devised as an unweighted (binary) adjacency matrix $A\in{\mathbb{R}}^{L \times L}$. The number of zero elements in the contact map/matrix is directly determined by the choice of the cutoff. With a higher cutoff, more non-zero edges would be preserved, and the computation time needed increases (Fig. S1A). To determine the optimal distance cutoff, we conducted an analysis using the ground truth labels of the 3,378 test set structures. We tested eight distance thresholds ranging from 6 Å to 13 Å and analyzed their impact on model performance metrics (Precision, Recall, and *F*_1_ score) (Fig. S1B). Given the ground truth labels of 3,378 structures in the test sets, precision was maximized within the 8–10 Å range, while recall peaked between 7 and 9 Å. Consequently, the 9 Å threshold yielded the highest overall *F*_1_ score (0.78). To investigate whether a threshold preference exists for specific enzyme classes, we evaluated the performance of 3rd- and 4th-digit predictions across the major EC categories. The 9 Å cutoff demonstrated consistent highest *F*_1_ score across the majority of classes (Table S1). Based on these findings, we selected 9 Å as the optimal distance cutoff for constructing the protein graphs. Furthermore, the average running time per epoch is illustrated in Fig. S1B. The average training time per epoch ranged from 44 seconds at 6 Å to 93 seconds at 13 Å. For our selected cutoff of 9 Å, the training time was approximately 60 seconds per epoch. Overall, we observed a linear increase in training time of approximately 11.3% for each 1 Å increment.

### Protein language model and graph network architecture

The model architecture of EC-LMGraph consists of (*i*) a protein language module to transform the input sequence into an efficient representation, and (*ii*) a graph network module for learning the structural representation of a protein. The protein language module is a transformer-based model ProtT5-XL-U50 [21] pre-trained with over 2,122 M protein sequences from BFD and refined with over 45 M protein sequences from UniProt database. To generate the feature representation of a protein, the amino acids are first tokenized and encoded into a numerical format, which is subsequently parsed by the protein language model. The last hidden layers of the ProtT5-XL-U50 model are selected to generate feature embeddings as the latter hidden layer typically captures a higher-level representation of the input protein sequence. The feature embeddings, together with the adjacency matrix and feature matrix, are fed into the graph network module to predict the enzyme functions.

GCNs have shown to be effective in graph feature learning tasks including protein function predictions [16], protein–protein interaction [55], and drug–target interactions [69, 70]. We conducted experiments on *five* different types of graph convolutions, including the extensively used graph convolutional layer (GCNConv) [20], graph attention (GATv2Conv) [29], hypergraph attention (HypergraphConv) [30], an efficient graph clustering convolution (ClusterGCNConv) [31], and local extremum convolution (LEConv) [32]. We analyze the effect of these convolutional layers on the classification performance of EC function and the number of catalytic sites identified. Considering that the LEConv achieved an exceptionally high identification rate on the catalytic sites and the ClusterGCNConv could attain a relatively higher *F*_1_ score, we selected the LEConv and ClusterGCNConv as the building block of our graph convolution module, followed by a mean pooling layer and fully-connected layers for classification purpose.

The graph convolutional layer LEConv takes the feature matrix ${{\bf X}}$ and the feature embeddings $L\in{\mathbb{R}}^{L \times 1024}$ from protein language model as a concatenated matrix ${{\bf H}}$ (i.e., $${{\bf H}} = [ {\begin{array}{*{20}{c}} {{\bf X}}&{{\bf L}} \end{array}} ]$$), and the contact map ${{\bf A}}$ as the inputs, computing the node embeddings for the next layer, ${H}^{( {l + 1} )}\in{\mathbb{R}}^{L \times {d}^{( {l + 1} )}}$:


(1) \begin{eqnarray*}
{{{\bf H}}}^{(l + 1)} = {\mathrm{LEConv}}\left( {{{\bf H}}{\mathrm{, }}{{\bf A}}} \right),
\end{eqnarray*}

where, ${d}^{(l)}$ is the dimension of node embeddings in layer *l*. With the LEConv, the importance of node *i* with respect to its neighborhood nodes $j \in \mathcal{N}(i)$ can be represented by the node embedding ${{\bf h}}_i^{(l + 1)}$ in the next layer, the node-wise formulation of Equation (1) as defined by Ranjan *et al*. [32] is


(2) \begin{eqnarray*}
{{\bf h}}_i^{(l + 1)} = {\mathrm{ReLU}}\left( {{{\bf h}}_i^{(l)}{{\bf W}}_1^{(l)} + \sum\limits_{j \in \mathcal{N}\left( i \right)} {{e}_{j,i}\left( {{{\bf h}}_i^{(l)}{{\bf W}}_2^{(l)} - {{\bf h}}_j^{(l)}{{\bf W}}_3^{(l)}} \right)} } \right),
\end{eqnarray*}

where, ${e}_{j,i}$ denotes the edge between node *i* and its’ neighbor node *j* in ${{\bf A}}$, and ${{\bf W}}_1^{( l )},{{\bf W}}_2^{( l )},{{\bf W}}_3^{( l )}\in {\mathbb{R}}^{{d}^{( l )} \times {d}^{( {l + 1} )}}$ are trainable weight matrices for layer *l*. The output after each LEConv undergoes a rectified linear activation function (ReLU) [71], i.e., ${\mathrm{ReLU}}( x ) = \max ( {x,0} )$, which sets the negative value to 0. Subsequently, the ClusterGCNConv outputs the node embeddings ${{{\bf H}}}^{(l + 1)}$ by taking the embeddings ${{{\bf H}}}^{(l)}$ updated by LEConv operators and the contact map ${{\bf A}}$:


(3) \begin{eqnarray*}
{{{\bf H}}}^{(l + 1)} = {\mathrm{ClusterGCNConv(}}{{{\bf H}}}^{(l)}{\mathrm{, }}{{\bf A}}{\mathrm{)}}.
\end{eqnarray*}

The formulation of Equation (3) defined by Chiang *et al*. [31] is


(4) \begin{eqnarray*}
{{{\bf H}}}^{( {l + 1} )} = {\mathrm{ReLU}}( {( {{{\bf \hat{A}}} + \lambda \cdot {\mathrm{diag}}( {{{\bf \hat{A}}}} )} ){{{\bf H}}}^{( l )}{{\bf W}}_1^{( l )} + {{{\bf H}}}^{( l )}{{\bf W}}_2^{( l )}} ),
\end{eqnarray*}

such that ${{\bf \hat{A}}} = {( {{{\bf D}} + {{\bf I}}} )}^{ - 1}( {{{\bf A}} + {{\bf I}}} )$, ${\mathrm{\lambda }}$is a diagonal enhancement value and two trainable weight matrices for layer *l* are denoted by ${{\bf W}}_1^{(l)},{{\bf W}}_2^{(l)}$. After the graph convolutional layers, a global mean pooling layer is applied on the resultant node embeddings to obtain a vector representation of the protein structure for graph classification. Such a pooling layer can ensure all protein graphs are represented by a fixed size vector independent of their number of nodes. Subsequent to the pooling layer, two fully connected layers with ReLU activation functions are used to compute the hidden representation from the pooled representation. At last, a fully connected layer is employed, where the number of output neurons corresponds to the total number of EC classes. To support multi-label prediction, each output neuron is activated by an independent sigmoid function, i.e., ${\mathrm{Sigmoid}}( x ) = 1/(1 + {e}^{ - x})$. This activation function maps the output of neurons to a value between 0 and 1, representing the probability belonging to the specific EC class. These independent sigmoid functions allow the model to simultaneously assign high probabilities to multiple classes.

The networks were supervised by minimizing the focal loss [36] function between the target class output *y* and the predicted probability *p*. Given a graph sample, the focal loss is defined as


(5) \begin{eqnarray*}
L = - {\left( {1 - {p}_i} \right)}^\gamma \log \left( {{p}_i} \right),
\end{eqnarray*}

where the probability ${p}_i$ is


(6) \begin{eqnarray*}
{p}_i = \left\{ \begin{array}{@{}l@{}} p\qquad\,\,\ {\mathrm{if }}\,\, y = 1\\ 1 - p\quad {\mathrm{otherwise}}. \end{array} \right.
\end{eqnarray*}

The focusing parameter $\gamma $ was set to 2, and the batch size was set to 20. Adam optimizer [72] were used to guide the neural network and update the weight parameters. The maximum number of training epochs was 500 and an early stopping criterion was set when training loss, validation loss and mean *F*_1_ score could not be further improved by 30 successive epochs. For ablation study, we evaluated the contribution of the protein language modules and the graph network architecture to the overall model performance. To achieve this, we trained additional models with specific modifications to isolate the impact of each component. First, we trained a model without the protein language module to assess its significance in extracting and enriching the protein sequence information. Second, we replaced the graph network module with common neural network architectures: (*i*) a fully connected network and (*ii*) a 1D-convolutional neural network. The framework was implemented on the PyTorch Geometric [73] (version 2.0.1) deep learning library, and all of the model training was executed with the use of NVIDIA GeForce RTX 3090 GPUs.

### Explainable annotation of catalytic sites

Highly conserved residues within the active domains of protein, namely catalytic sites, can be identified to attribute the enzyme function. Predicting the residues that are directly involved in the catalytic functions could facilitate the design of mutagenesis experiment, thus confirming its enzymatic activity. The 3D conformations and groups of amino acids involved in the catalytic activities become highly relevant features for graph network models to learn and predict the corresponding EC classes. A method to explain the model prediction and quantify the importance of the input amino acid nodes could pinpoint a promising target set of residues in charge of the ultimate enzymatic functioning. Therefore, the activation maps of our network, which are expected to capture the structural features of functional regions, can be post-processed to quantify the learned feature map values onto the input amino acid nodes.

Quantitative evaluation was performed to assess the correspondence between the model-predicted catalytic residues and actual catalytic sites. M-CSA dataset [25] is chosen to derive a set of actual catalytic sites, considering that their catalytic site residues annotations have been experimentally validated. Given a positively predicted protein graph, we computed the activation values using various method, including saliency map (Saliency) [40], multiplies gradient with respect to input (InputXGradient) [41], guided backpropagation (GuidedBackprop) [42], and deconvolution (Deconvolution) [43]. Taking the top 10% residue positions as model-predicted catalytic residues, the saliency method was chosen given that a stronger correlation was observed between the model-predicted catalytic sites and the annotated catalytic sites. Specifically, the Saliency map was computed by assigning relevance scores to the input graph nodes (residues) based on the partial derivative of the model’s output. Next, these scores were aggregated as activation values by taking the absolute value of the gradient, which represents the strength of the activation associated with each input node. The activation values were then normalized into a scale of [0,1] in order to generate the final Saliency map. To evaluate the correspondence, function-specific activation values were computed to identify the top 10% predicted residues. A window size of 0 to ±5 was applied to assess the number of actual catalytic sites that can be covered. The prediction is also evaluated versus random coincidences, the same numbers of amino acid positions (i.e., 10% of the protein length) were randomly drawn across the amino acid chain. This proportional sampling method could reduce size-dependent effects that arise from proteins having different sizes and lengths, allowing for comparison across all proteins. The mean Euclidean distance between the predicted residue sites and the nearest M-CSA sites was calculated to illustrate whether the model-predicted residues would have a lower mean distance than the random drawn. This process is repeated 1,000 times, and the event counts are plotted. The predicted residues were mapped to the PDB structures upon the amino acid position, and then visualized using UCSF Chimera [74].

## Availability of source code and requirements

Project name: EC-LMGraph

Project homepage: https://github.com/ngyuilun/EC-LMGraph

Operating system: Platform independent

Programming language: Python

Other requirements: Listed in the *requirements.txt* file provided in the repository

License: MIT license

RRID:SCR_027317

## Additional files

**Supplementary Fig. S1:** Building protein graph representation and dependence of prediction performance on distance cut-off. (A) Protein graphs obtained with different distance cut-offs for the same example shown in Fig. 1A. (B) Dependence of EC-LMGraph performance (Precision, Recall, *F*_1_) and training time on distance cut-offs ranging from 6 to 13.

**Supplementary Fig. S2:** (A) Enzyme commission number prediction performance of different graph convolutional layers. (B) Number of M-CSA catalytic residues covered by residues with top 5% or 10% activation values using different graph convolutional layers.

**Supplementary Fig. S3:** (A) Prediction performance of EC-LMGraph optimized by binary cross-entropy loss compared to Focal Loss with varying focusing parameters ($\gamma = 1,\ 2,\ 3,\ 4$). (B) Comparison of the prediction performance of EC-LMGraph, fully connected network, and convolutional neural network on the learning of the protein language model. The performance of EC-LMGraph trained with ProtBert, with ESM-2, and without the protein language model is also included for comparison. (C) Accuracy, specificity, and Matthews correlation coefficient (MCC) of EC-LMGraph for the prediction of enzyme commission (EC) main class, subclass, sub-subclass, and sub-sub-subclass numbers. (D) Computation time of the various compared methods. The prediction framework was used to predict EC numbers for 100, 200, 500, and 1,000 randomly selected proteins.

**Supplementary Fig. S4:** Total number of M-CSA catalytic residues covered by residues with top 5% or 10% activation values using different explainability methods with EC-LMGraph.

**Supplementary Fig. S5:** (A) The ratio of the number of correctly predicted sites to the total number of predicted catalytic residues, evaluated across a window size range of ±1 to ±5. (B) Total number of catalytic residues annotated in the Mechanism and Catalytic Site Atlas (M-CSA) that are covered within the window sizes from 3 Å to 7 Å.

**Supplementary Fig. S6:** Performance of EC-LMGraph predictions relative to AlphaFold2 structural confidence (pLDDT). The performance was evaluated for High (>90), Medium (70–90), and Low (<70) pLDDT groups across all EC hierarchy levels. Error bars represent 95% confidence intervals derived from bootstrap resampling.

**Supplementary Fig. S7:** Catalytic sites prediction for Parkinson’s disease-related enzymes using DeepFRI–Grad-CAM. (A) Ubiquitin carboxyl-terminal hydrolase isozyme L1, (B) Serine protease HTRA2, (C) Protein deglycase DJ-1, and (D) Parkin.

**Supplementary Table S1:** Stratified evaluation of EC-LMGraph prediction performance (*F*_1_ score) across major enzyme commission (EC) classes using distance cutoffs ranging from 6 Å to 13 Å.

**Supplementary Table S2:** Distribution of training samples across the four hierarchical levels of the EC classification.

**Supplementary Table S3:** Performance comparison of catalytic site prediction between EC-LMGraph and DeepFRI.

**Supplementary Table S4:** EC-LMGraph prediction performance on AlphaFold2-predicted structures.

## Competing interests

The authors declare no competing interests.

## Supplementary Material

giag056_Supplemental_File

giag056_Authors_Response_To_Reviewer_Comments_original_submission

giag056_GIGA-D-25-00310_original_submission

giag056_GIGA-D-25-00310_revision_1

giag056_Reviewer_1_Report_original_submission Reviewer 1 -- 9/20/2025

giag056_Reviewer_2_Report_original_submission Reviewer 2 -- 11/25/2025

giag056_Reviewer_2_Report_revision_1 Reviewer 2 -- 4/5/2026

## Data Availability

The protein structure data and EC number annotations described in this manuscript were downloaded from wwPDB [75] and UniProt [76], respectively. The enzyme catalytic residues are based on annotations documented in M-CSA [77]. The AlphaFold2-predicted structures were downloaded from AlphaFold DB [78]. The post-processed training and test datasets, source code, models with their network weight, as well as all the entries of prediction results are freely available in the EC-LMGraph repository [28].
