# Supplementary material for: Accurate proteome-wide prediction of enzymes and catalytic sites using graph deep learning and protein language model
Source: Gigascience. 2026 May 13;15:giag056. doi: 10.1093/gigascience/giag056 (PMC13217607; doi:10.1093/gigascience/giag056)
Supplement: giag056_Supplemental_File [file giag056_supplemental_file.pdf]

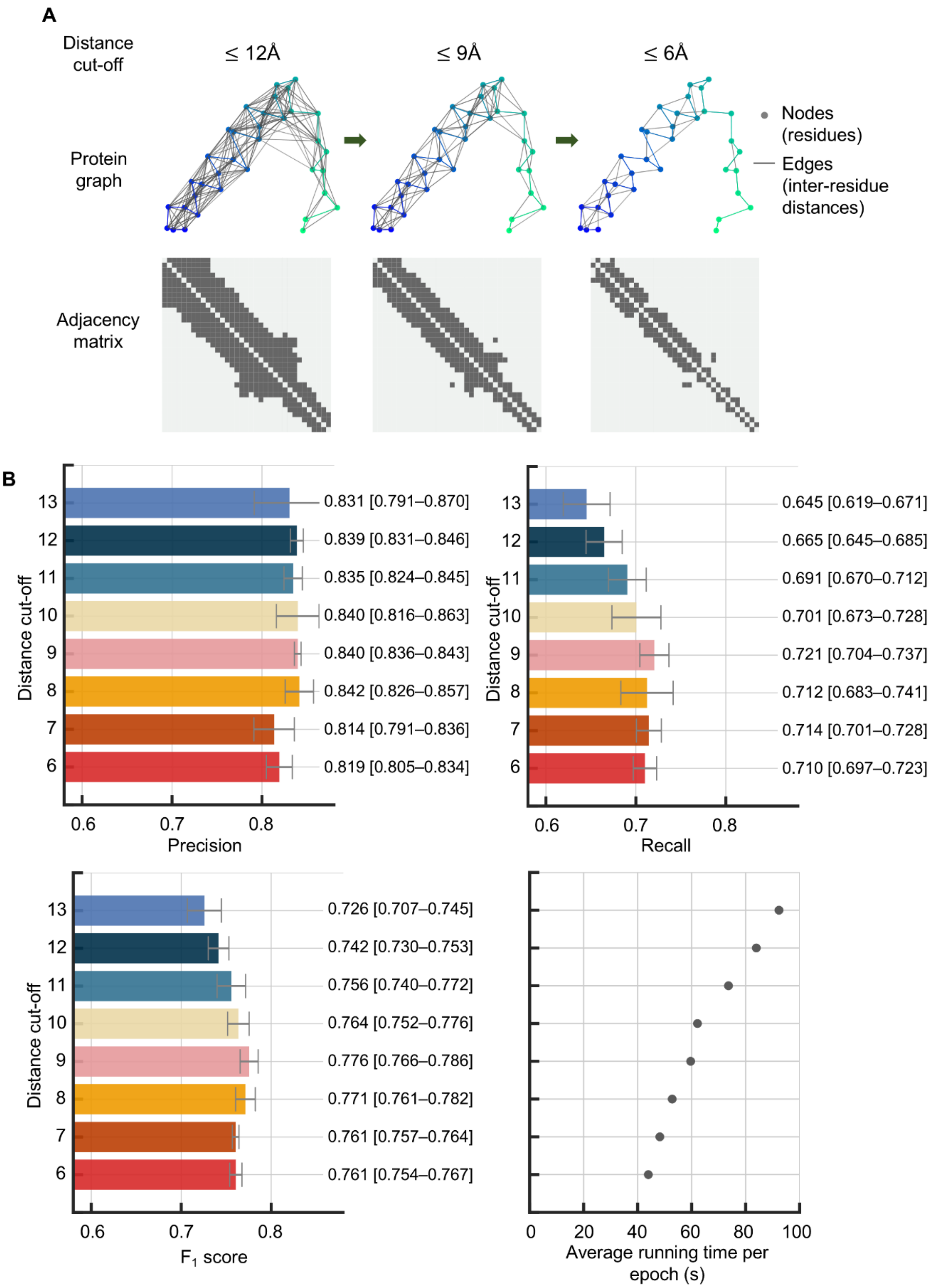

Fig. S1: Building protein graph representation and dependence of prediction performance on distance cut-off. (A) Protein graphs obtained with different distance cut-offs for the same example shown in **Fig. 1A**. **(B)** Dependence of EC-LMGraph performance (Precision, Recall, F_1) and training time on distance cut-offs ranging from 6 to 13.

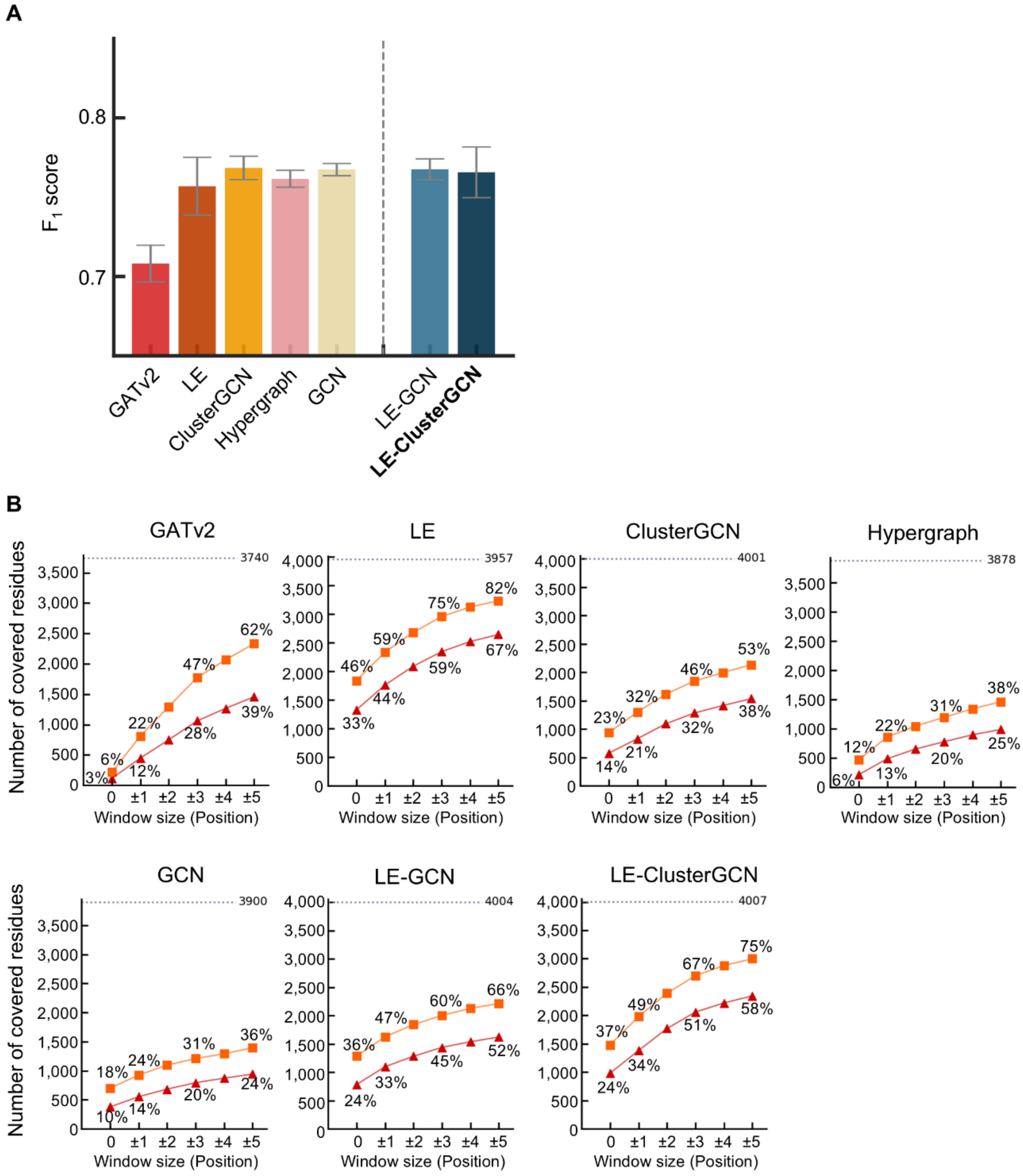

Fig. S2: (A) Enzyme commission number prediction performance of different graph convolutional layers. **(B)** Number of M-CSA catalytic residues covered by residues with top 5% or 10% activation values using different graph convolutional layers.

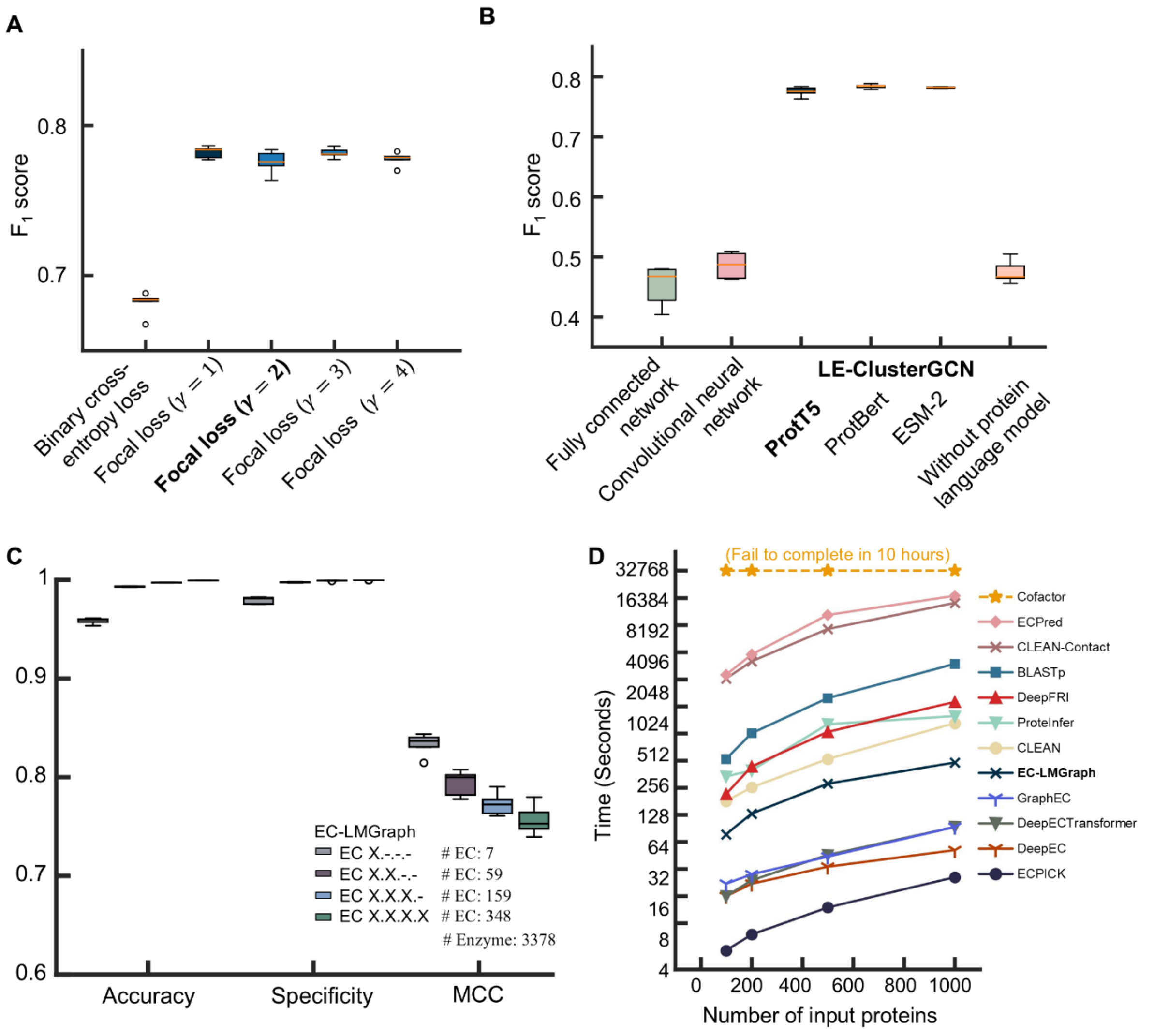

Fig. S3: (A) Prediction performance of EC-LMGraph optimized by Binary Cross-Entropy loss compared to Focal Loss with varying focusing parameters ($\gamma = 1, 2, 3, 4$). **(B)** Comparison of the prediction performance of EC-LMGraph, fully connected network, and convolutional neural network on the learning of the protein language model. The performance of EC-LMGraph trained with ProtBert, with ESM-2, and without the protein language model is also included for comparison. **(C)** Accuracy, specificity and Matthews correlation coefficient (MCC) of EC-LMGraph for the prediction of Enzyme Commission (EC) main class, subclass, sub-subclass, and sub-sub-subclass numbers. **(D)** Computation time of the various compared methods. The prediction framework was used to predict EC numbers for 100, 200, 500, and 1,000 randomly selected proteins.

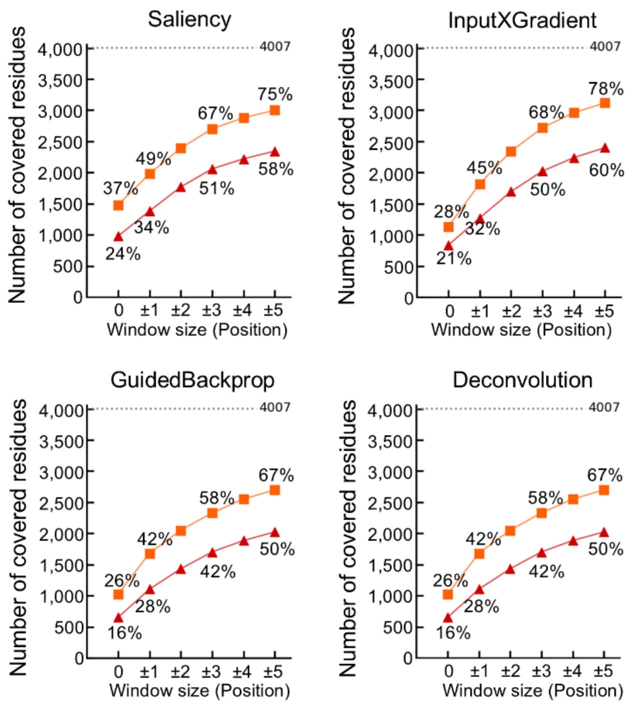

Fig. S4: Total number of M-CSA catalytic residues covered by residues with top 5% or 10% activation values using different explainability methods with EC-LMGraph.

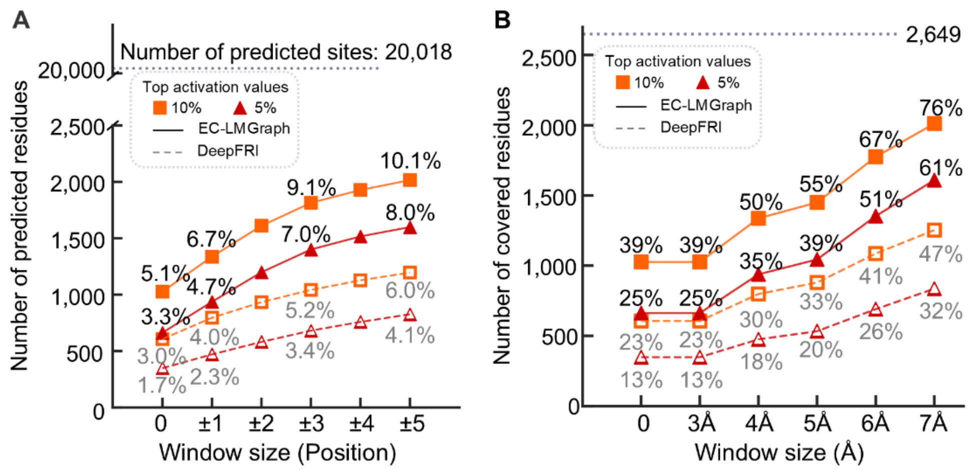

Fig. S5: (A) The ratio of the number of correctly predicted sites to the total number of predicted catalytic residues, evaluated across a window size range of ±1 to ±5. **(B)** Total number of catalytic residues annotated in the Mechanism and Catalytic Site Atlas (M-CSA) that are covered within the window sizes from 3Å to 7Å.

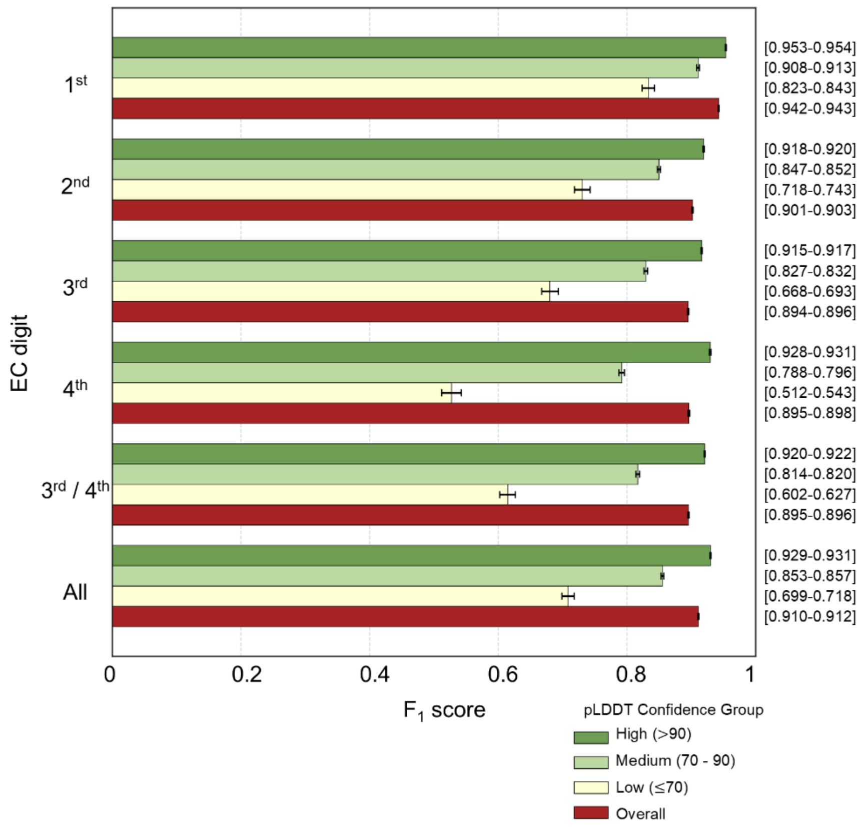

Fig. S6: Performance of EC-LMGraph predictions relative to AlphaFold2 structural confidence (pLDDT). The performance was evaluated for High (>90), Medium (70–90), and Low (<70) pLDDT groups across all EC hierarchy levels. Error bars represent 95% confidence intervals derived from bootstrap resampling.

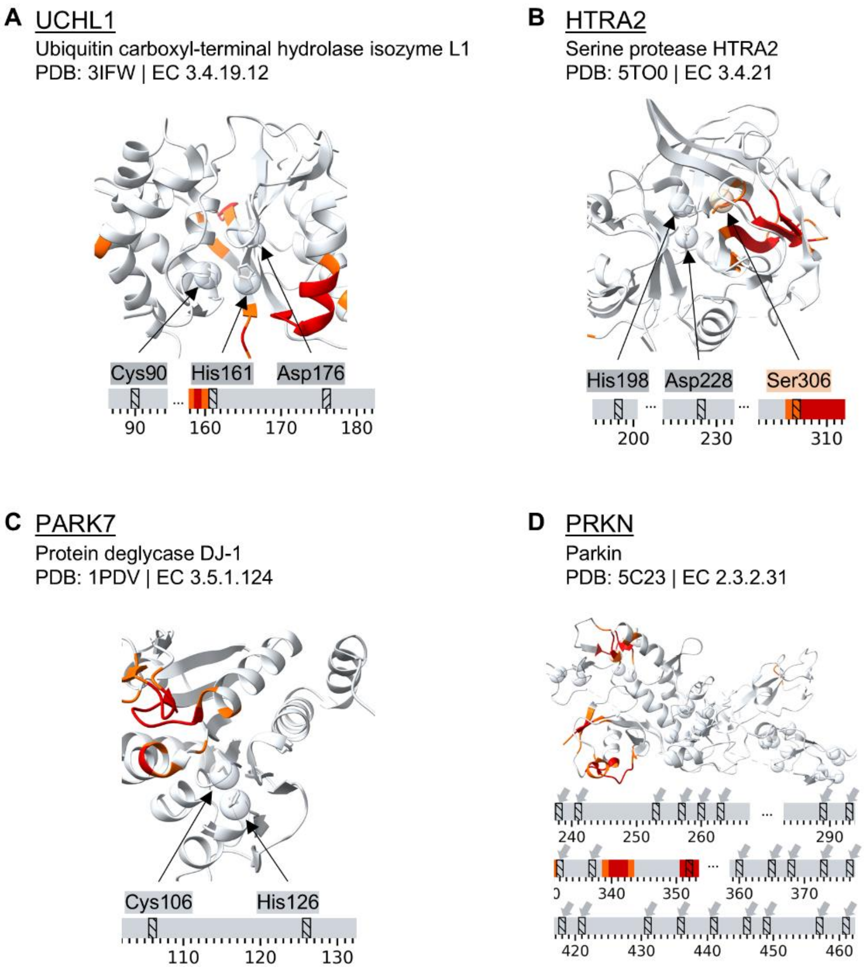

Fig. S7: Catalytic sites prediction for Parkinson's disease-related enzymes using DeepFRI–Grad-CAM. (A) Ubiquitin carboxyl-terminal hydrolase isozyme L1, **(B)** Serine protease HTRA2, **(C)** Protein deglycase DJ-1, and **(D)** Parkin.

Table S1: Stratified evaluation of EC-LMGraph prediction performance (F_1 score) across major Enzyme Commission (EC) classes using distance cutoffs ranging from 6Å to 13Å.

	3rd digit						
Distance Cutoff	EC 1.x.x.-	EC 2.x.x.-	EC 3.x.x.-	EC 4.x.x.-	EC 5.x.x.-	EC 6.x.x.-	EC 7.x.x.-
6	0.689	0.804	0.768	0.619	0.663	0.820	0.797
7	0.686	0.807	0.764	0.654	0.662	0.833	0.797
8	0.694	0.811	0.768	0.647	0.671	0.817	0.834
9	0.705	0.815	0.770	0.670	0.684	0.840	0.849
10	0.695	0.807	0.775	0.646	0.641	0.830	0.822
11	0.686	0.808	0.760	0.639	0.622	0.819	0.823
12	0.673	0.793	0.745	0.610	0.615	0.831	0.809
13	0.637	0.776	0.741	0.603	0.601	0.811	0.799

	4th digit						
Distance Cutoff	EC 1.x.x.x	EC 2.x.x.x	EC 3.x.x.x	EC 4.x.x.x	EC 5.x.x.x	EC 6.x.x.x	EC 7.x.x.x
6	0.806	0.765	0.713	0.750	0.875	0.888	0.694
7	0.806	0.771	0.703	0.796	0.856	0.864	0.686
8	0.818	0.780	0.724	0.760	0.884	0.870	0.650
9	0.843	0.786	0.727	0.767	0.871	0.910	0.706
10	0.809	0.763	0.709	0.751	0.863	0.882	0.673
11	0.798	0.757	0.705	0.745	0.812	0.891	0.644
12	0.751	0.754	0.693	0.703	0.781	0.794	0.623
13	0.704	0.735	0.680	0.619	0.743	0.751	0.621

*The highest F_1 score is highlighted in red, and the second highest is highlighted in green.

Table S2: Distribution of training samples across the four hierarchical levels of the EC classification

EC Level	Description	Number of unique classes	Total training samples	Number of samples per class	Median samples per class	Classes with ≤ 10 samples	Classes with ≤ 50 samples
Level 1	Main Class (e.g., EC 1.-.-.-)	7	12,230	1747.1	906	0 (0.0%)	0 (0.0%)
Level 2	Subclass (e.g., EC 1.1.-.-)	61	12,261	201	76	6 (9.8%)	26 (42.6%)
Level 3	Sub-subclass (e.g., EC 1.1.1.-)	175	11,886	67.9	28	41 (23.4%)	117 (66.9%)
Level 4	Substrate (e.g., EC 1.1.1.1)	561	7,497	13.4	8	380 (67.7%)	541 (96.4%)

Table S3: performance comparison of catalytic site prediction between EC-LMGraph and DeepFRI

	EC-LMGraph	DeepFRI
Accuracy	0.896	0.892
Precision	0.050	0.029
Recall	0.390	0.224
Specificity	0.903	0.900
F1 Score	0.088	0.051
MCC	0.109	0.047
Macro PR-AUC	0.220	0.133

Table S4: EC-LMGraph prediction performance on AlphaFold2-predicted structures.

	Species	Number of known enzymes	Number of true positives	Precision	Recall	F_1 score	MCC
Model Organism Proteomes	*H. sapiens*	3,115	2,139	79%	69%	73%	74%
	A. thaliana	2,769	1,882	78%	68%	73%	73%
	M. musculus	2,457	1,753	83%	71%	77%	77%
	R. norvegicus	1,387	1,002	84%	72%	78%	78%
	S. cerevisiae	1,029	625	87%	61%	72%	73%
	O. sativa	854	571	81%	67%	73%	73%
	D. discoideum	732	529	85%	72%	78%	78%
	S. pombe	721	517	82%	72%	77%	77%
	C. elegans	688	464	74%	67%	71%	71%
	E. coli	598	502	86%	84%	85%	85%
	D. melanogaster	584	388	78%	66%	72%	72%
	D. rerio	415	294	81%	71%	76%	76%
	M. jannaschii	227	175	80%	77%	78%	78%
	C. albicans	207	157	92%	76%	83%	83%
	Z. mays	153	130	86%	85%	85%	85%
	G. max	70	61	75%	87%	81%	81%
Global Health Proteomes	*M. tuberculosis*	466	360	89%	77%	83%	83%
	S. typhimurium	393	343	90%	87%	89%	89%
	P. aeruginosa	368	330	92%	90%	91%	91%
	H. influenzae	353	311	92%	88%	90%	90%
	M. leprae	251	209	95%	83%	89%	89%
	S. dysenteriae	220	203	93%	92%	92%	92%
	S. aureus	213	185	93%	87%	90%	90%
	H. pylori	208	181	97%	87%	92%	92%
	S. pneumoniae	181	164	99%	91%	95%	95%
	C. jejuni	181	166	98%	92%	95%	95%
	N. gonorrhoeae	161	153	100%	95%	97%	97%
	P. falciparum	90	64	77%	71%	74%	74%
	S. mansoni	25	25	96%	100%	98%	98%
	T. brucei	20	10	83%	50%	63%	65%
	A. capsulatus	16	7	78%	44%	56%	58%
	T. cruzi	14	7	78%	50%	61%	62%
	B. malayi	13	10	91%	77%	83%	84%
	P. lutzii	13	8	89%	62%	73%	74%
	O. volvulus	8	8	100%	100%	100%	100%
	L. infantum	7	4	100%	57%	73%	76%
	E. faecium	6	5	71%	83%	77%	77%
	W. bancrofti	1	0	0%	0%	0%	0%
	M. ulcerans	1	1	100%	100%	100%	100%
