# Supplementary material for: Accurate proteome-wide prediction of enzymes and catalytic sites using graph deep learning and protein language model
Source: Gigascience. 2026 May 13;15:giag056. doi: 10.1093/gigascience/giag056 (PMC13217607; doi:10.1093/gigascience/giag056)
Supplement: giag056_Authors_Response_To_Reviewer_Comments_original_submission [file giag056_authors_response_to_reviewer_comments_original_submission.pdf]

Author's Response To Reviewer Comments

Authors' Responses
GIGA-D-25-00310
We would like to thank the Editor-in-Chief, Associate Editor, and the reviewers for their constructive suggestions and criticism. The comments provided by the reviewers are very resourceful and helpful. We have included more data to address the concerns raised and made changes to parts of the manuscript where we or the reviewers felt more clarity or detail was required. All the points are addressed individually in detail below.

Reviewer 1:
Comment:
The EC-LMGraph framework proposed in this paper innovatively combines protein language models with graph convolutional networks, realizing the collaborative optimization of 3rd/4th-level EC number prediction for enzymes and catalytic site localization. Meanwhile, it expands the application scenarios of the whole proteome through AlphaFold2-predicted structures. The study features a rigorous design, comprehensive verification dimensions, and a good balance between interpretability and computational efficiency. Compared with existing methods, it shows obvious advantages and holds significant reference value for enzyme function annotation and experimental design.
However, the paper still has room for improvement in terms of the completeness of methodological details, the depth of result analysis, and the comparison with the latest methods. Further experimental evidence and logical explanations need to be supplemented to enhance the persuasiveness of the conclusions.
Answer:
Many thanks for your recommendation and insightful comments, to which we explained as follows, along with the modifications in our new version of manuscript.

R1-1 Comment:
Comment 1:
1. The paper states that "9Å is the optimal distance threshold", yet only mentions the optimization range (6-13Å) in Supplementary Fig. S1B. The following information is not clearly specified in the main text: The specific impact of different thresholds (e.g., 6Å, 9Å, 12Å) on the prediction performance of 3rd/4th-level EC numbers (such as differences in F1 score, precision, and recall)
Answer:
We appreciate the reviewer's constructive feedback regarding the rationale for selecting the distance threshold. We have expanded our analysis and revised the manuscript to clarify how these thresholds affect prediction performance.
We extend the analysis in Fig. S1B to illustrate the trends of Precision and Recall alongside the F1 score across the optimization range (6–13Å). Our results show that the model maintains high precision (>0.84) across the 8–10Å range, peaking at 8Å (0.842) and remaining highly robust at 9Å and 10Å (0.840). In terms of the recall, the performance is superior in the 7–9Å range, with the 9Å threshold achieving the highest recall of 0.721. Balancing both metrics, we find that the 9Å threshold yields the highest overall F1 score (~0.776). This indicates that 9Å effectively captures the necessary local structural environment for enzyme function predictions without inducing the excessive noise attributed by the larger distance thresholds. In the revised manuscript, we have updated Fig. S1B to show how F1 score, precision, and recall vary across the selected distance cutoffs.
Comment 2:
particularly whether there exists a threshold preference when predicting different major EC classes (e.g., EC 3 hydrolases, EC 7 translocases).
Answer:
To address whether a threshold preference exists for specific enzyme classes, we have analyzed the optimal threshold for the 3rd- and 4th-digit prediction across all seven major EC classes. At the 3rd-digit level, the 9Å threshold yields the highest F1 score in six of the seven classes, with Hydrolases (EC 3) being the only exception by slightly favoring a 10Å cutoff. Similarly, the 9Å threshold remains optimal for five of the seven classes at the 4th-digit level. Specifically, Lyases (EC 4) and Isomerases (EC 5) show minor

preferences for 7Å and 8Å, respectively. Despite these minor variations, the 9Å cutoff offers the most robust prediction accuracy across the classes. We have added Table S1 to illustrate these trends across the metrics and the seven major EC classes.

Comment 3:

Overall the author should comment on insufficient rationale for distance threshold optimization in protein graph construction; The quantitative relationship between thresholds and computational efficiency (e.g., a 1Å increase results in an 11.3% rise in computation time, but is the corresponding performance improvement significant?).

Answer:

Regarding computational efficiency, we recognize that the original text may have implied that computational time was a primary consideration in the cutoff selection. We would like to clarify that the distance cutoff was determined solely by performance (F1 score), but the computational time serves as a secondary measurable outcome only. In the revised manuscript, we have reorganized the presentation to make this priority explicit.

The aforementioned revisions are listed as follows:

Page 4, 1st paragraph:

We analyzed the trade-offs between precision and recall across thresholds ranging from 6Å to 13Å (Fig. S1B). We observed that precision remained consistently high (>0.84) for cutoffs between 8Å and 10Å. Regarding recall, the model's ability to retrieve true positives was superior when the cutoff is between 7–9Å, with the 9Å cutoff achieving the maximum recall of 0.72. Consequently, the 9Å cutoff yielded the highest overall F1 score (0.78), effectively balancing the capture of relevant structural features against the inclusion of noise. To determine whether a threshold preference exists for specific enzyme classes, we further stratified the performance of 3rd- and 4th-digit predictions across major EC categories (Table S1). A distance cutoff of 9Å consistently demonstrated robustness across diverse functional categories. At the 3rd-digit level, the 9Å threshold is optimal for six of the seven classes; Hydrolases (EC 3) is the sole exception, slightly favoring a 10Å cutoff. Regarding 4th-level predictions, the 9Å cutoff remains the top performer for five classes, while Lyases (EC 4) and Isomerases (EC 5) exhibit minor preferences for 7Å and 8Å, respectively. Despite these slight variations, the 9Å threshold provides the most consistent and accurate performance across the majority of enzyme functions. Hence, we empirically chose the 9Å cutoff as optimal for defining residue contacts to effectively learn graph structures. Through these preprocessing steps, each protein structure was modeled as a node property-preserved, thresholded, and undirected graph.

Page 16, 2nd paragraph:

To determine the optimal distance cutoff, we conducted an analysis using the ground truth labels of the 3,378 test set structures. We tested eight distance thresholds ranging from 6Å to 13Å and analyzed their impact on model performance metrics (Precision, Recall, and F1 score) (Fig. S1B). Given the ground truth labels of 3,378 structures in the test sets, precision was maximized within the 8–10Å range, while recall peaked between 7–9Å. Consequently, the 9Å threshold yielded the highest overall F1 score (0.78). To investigate whether a threshold preference exists for specific enzyme classes, we evaluated the performance of 3rd- and 4th-digit predictions across the major EC categories. The 9Å cutoff demonstrated consistent highest F1 score across the majority of classes (Table S1). Based on these findings, we selected 9Å as the optimal distance cutoff for constructing the protein graphs. Furthermore, the average running time per epoch is illustrated in Fig. S1B. The average training time per epoch ranged from 44 seconds at 6Å to 93 seconds at 13Å. For our selected cutoff of 9Å, the training time was approximately 60 seconds per epoch. Overall, we observed a linear increase in training time of approximately 11.3% for each 1Å increment.

Table S1:

Table S1: Stratified evaluation of EC-LMGraph prediction performance (F1 score) across major Enzyme Commission (EC) classes using distance cutoffs ranging from 6Å to 13Å.

	3rd digit						
Distance Cutoff	EC 1.x.x.-	EC 2.x.x.-	EC 3.x.x.-	EC 4.x.x.-	EC 5.x.x.-	EC 6.x.x.-	EC 7.x.x.-
6	0.689	0.804	0.768	0.619	0.663	0.820	0.797
7	0.686	0.807	0.764	0.654	0.662	0.833	0.797
8	0.694	0.811	0.768	0.647	0.671	0.817	0.834
9	0.705	0.815	0.770	0.670	0.684	0.840	0.849
10	0.695	0.807	0.775	0.646	0.641	0.830	0.822

11	0.686	0.808	0.760	0.639	0.622	0.819	0.823
12	0.673	0.793	0.745	0.610	0.615	0.831	0.809
13	0.637	0.776	0.741	0.603	0.601	0.811	0.799

| 4th digit

Distance Cutoff	EC 1.x.x.x	EC 2.x.x.x	EC 3.x.x.x	EC 4.x.x.x	EC 5.x.x.x	EC 6.x.x.x	EC 7.x.x.x
6	0.806	0.765	0.713	0.750	0.875	0.888	0.694
7	0.806	0.771	0.703	0.796	0.856	0.864	0.686
8	0.818	0.780	0.724	0.760	0.884	0.870	0.650
9	0.843	0.786	0.727	0.767	0.871	0.910	0.706
10	0.809	0.763	0.709	0.751	0.863	0.882	0.673
11	0.798	0.757	0.705	0.745	0.812	0.891	0.644
12	0.751	0.754	0.693	0.703	0.781	0.794	0.623
13	0.704	0.735	0.680	0.619	0.743	0.751	0.621

*The highest F1 score is highlighted in red, and the second highest is highlighted in green.

R1-2 Comment:
2. The author directly adopts ProtT5-XL-U50 as the sequence feature extraction module, yet fails to explain why other mainstream pLMs are not chosen. It is advisable to add comparative experiments of pLMs (including at least 1-2 mainstream models) or clarify the core basis for selecting ProtT5 in the discussion section.
Answer:
We thank the reviewer for this insightful suggestion. To address this, we have conducted a comparative experiment using two additional mainstream protein language models (pLMs), which are ProtBert and ESM-2. We also compare these two with our original ProtT5-XL-U50. Separate iterations of EC-LMGraph are trained using embeddings from every respective model while keeping the LE-ClusterGCN architecture constant across all configurations.
As shown in the updated Fig. S3B, all the three models achieve highly similar results, with F1 scores consistently clustering around 0.78. These findings indicate that the EC-LMGraph framework is highly robust, capable of leveraging rich sequence representations effectively from these state-of-the-art pLMs. Consequently, while ProtBert and ESM-2 are valid alternatives, we retained ProtT5-XL-U50 as our primary sequence feature extractor. It is convincing that the choice of pLM does not limit the model's predictive capability. We have updated the Results section to discuss the resultant robustness based on the comparable data.
In our revised manuscript, we have included evaluation of our framework using embeddings from other mainstream protein language models, namely ProtBert and ESM-2. We updated Fig. S3B to demonstrate that all three models achieved highly similar F1 scores, confirming the framework's robustness, thus justifying our selection of ProtT5 without limiting the model's predictive capabilities.
The aforementioned revisions are listed as follows:
Page 6, 1st paragraph:
To assess the influence of mainstream protein language models on enzyme prediction, we trained LE-ClusterGCN architecture using embeddings from ProtT5-XL-U50 [21], ProtBert [36], and ESM-2 [22]. All three models demonstrated comparable performance, yielding robust F1 scores ranging from 0.77 to 0.78 (Fig. S3B). The overlapping confidence intervals (ProtT5: 0.76–0.79; ProtBert: 0.78–0.79; ESM-2: 0.78–0.78) indicate that the prediction accuracy is not heavily dependent on the specific pLM architecture. This consistency suggests that LE-ClusterGCN architecture effectively integrates structural graph features with high-dimensional sequence embeddings, regardless of whether the embeddings are derived from encoder-only (ProtBert, ESM-2) or encoder-decoder (ProtT5) architectures. Given this robustness, we chose ProtT5 embeddings as the representative sequence feature extractor for the subsequent analysis.

R1-3 Comment:
3. The EC prediction need to compare with CLEAN-Contact, the newly published paper.
Answer:
Many thanks for your kind recommendation. To conduct a comprehensive comparison, new experiments with CLEAN-Contact are included in our benchmarking analysis. We identify newly annotated protein structures and sequences in the UniProtKB database 2025_02 (April 2025) that were not included in the

previous 2022_01 release (February 2022). On these newly annotated structures, CLEAN-Contact achieved F1 scores ranging from 0.43 to 0.60. These scores are higher than the original CLEAN method (0.34–0.54), but also consistently lower than EC-LMGraph (0.49–0.76) across all the sequence similarity levels. When it is tested on the newly annotated sequence set, EC-LMGraph attains the highest F1 scores, 0.90, 0.85 and 0.82, respectively, for predicting the 1st-, 2nd-, and 3rd-digit EC numbers. Up to the 4th-digit prediction, CLEAN-Contact achieves the highest F1 score = 0.74.
These results point to a complementary distinction between the two frameworks. EC-LMGraph's use of precise three-dimensional structural graphs confers a distinct advantage in capturing the core catalytic mechanisms that define the 1st-, 2nd-, and 3rd-digit EC classes. In contrast, CLEAN-Contact, by leveraging massive sequence databases, appears highly effective at capturing the specificities of 4th-digit predictions. It is worth noting that with a protein language module pre-trained on large sequence databases, EC-LMGraph achieves these results by learning through a substantially smaller annotated training set, comprising only proteins with experimentally validated structures. The presented EC-LMGraph remains highly competitive, even when the numbers of samples in the 4th-digit classes are especially small.
In our revised manuscript, we present the new benchmarking experiments that compare our model against the newly published CLEAN-Contact framework. Fig. 2C is updated to illustrate the performance on the newly annotated enzyme structures. The one on the newly annotated enzyme sequences is also depicted in Fig. 2D–F.
The specific additions to the revised manuscript are as follows:
Page 7, 2nd paragraph:
We benchmarked our models against the current state-of-the-art sequence-based and structure-based methods, namely CLEAN [4], CLEAN-Contact [5] and DeepFRI [16], respectively (Fig. 2C).
Page 7, 2nd paragraph:
Specifically, EC-LMGraph achieved F1 scores of 0.76 at 95% similarity cut-off, and 0.49 at 30% similarity cut-off, CLEAN-Contact achieved F1 scores ranging from 0.43 to 0.60, while CLEAN and DeepFRI achieved F1 scores of 0.34¬–0.54 and 0.23–0.48, respectively (Fig. 2C).
Page 7, 3rd paragraph:
We then compared the performance of EC-LMGraph against eleven other methods, including the sequence alignment method (BLASTp) [3], sequence-based deep learning methods (CLEAN [4], CLEAN-Contact [5], DeepECTransformer [6], ProteInfer [7], ECPred [8], DeepEC [9], GraphEC [10], and ECPICK [11]), and structure-based methods (DeepFRI [16] and COFACTOR [15]). In the overall combined 3rd/4th-level EC evaluation, CLEAN-Contact and DeepECTransformer showed strong performance with F1 scores of ~0.78 and ~0.70, respectively, while EC-LMGraph followed closely behind (~0.69) (Fig. 2D). When breaking down the evaluation by independent hierarchical levels, EC-LMGraph consistently outperformed the other methods on broader functional classes, attaining the highest F1 scores for predicting the 1st-, 2nd-, and 3rd-digit EC classes (0.90, 0.85, and 0.82, respectively) (Fig. 2E). Up to the 4th-digit prediction, CLEAN-Contact achieved the highest F1 score (0.74), followed by DeepECTransformer (0.65). EC-LMGraph achieved results similar to those of BLASTp, CLEAN, and ProteInfer, with scores in the ~0.53–0.55 range (Fig. 2E). The relative performances remained consistent across sequence similarity levels (Fig. 2F). These results suggest that utilizing precise 3D structural graphs gives EC-LMGraph a distinct advantage in capturing the core catalytic mechanisms that define the 1st-, 2nd-, and 3rd-digit EC classes. In contrast, sequence-based methods utilize massive databases to access a substantial volume of sequence examples, effectively capturing the fine-grained substrate specificities characterizing 4th-digit EC classes.

R1-4 Comment:
4. Lack of Basis for Parameter Setting of Focal Loss. In the paper, the focusing parameter γ is fixed at 2, yet no explanation is provided for why this value is chosen. It is recommended to supplement the comparison of F1 scores under different γ values and clarify how $\gamma=2$ optimally addresses the sample imbalance issue in 4th-level EC prediction.
Answer:
We thank the reviewer for pointing out the need to justify the selection of the focusing parameter. To address this concern, we have conducted additional training experiments to evaluate the impact of Focal Loss parameters (γ) across values ranging from 1 to 4. We have also utilized the Binary Cross-Entropy (BCE) loss as a baseline for this comparison.
The model optimized with the baseline BCE loss attains an F1 score of ~0.68. By introducing the focal loss, we can accomplish a substantial improvement across all γ values tested, such that the F1 scores can

consistently reach up to ~0.77–0.78. As the performance variations among these parameters are marginal, we have selected the standard setting of γ = 2 in our further analysis, which has been established as an effective default in the original focal loss literature and is consistent with common practice in tasks involving class imbalance. This setting was applied uniformly across all subsequent analysis.
In our revised manuscript, we have updated Fig. S3A to illustrate these comparisons.
The aforementioned revisions are listed as follows:
Page 5, 2nd paragraph:
We evaluated the model's performance using focusing parameters (γ) ranging from 1 to 4 and compared these results to the baseline binary cross-entropy (BCE) loss within a five-fold cross-validation setting. The model optimized with baseline BCE loss attained an F1 score of ~0.68. By introducing the focal loss, we can accomplish a substantial improvement across all γ values tested, such that the F1 scores can consistently reach up to ~0.77–0.78 (Fig. S3A). All tested γ values yield consistently high scores across folds, suggesting that the focal loss mechanism itself drives the performance gain. As the performance variations among the tested γ parameters were marginal, we adopted the standard setting of γ=2.

R1-5 Comment:
5. The paper mentions that "on AlphaFold2-predicted structures, the F1 score for 4th-level EC prediction is 0.54, which is lower than that of BLASTp (0.55) and ProteInfer (0.54)", yet it fails to conduct an in-depth analysis of the reasons behind this phenomenon.
Answer:
Many thanks for your kind recommendation and comments.
Relative to sequence-based methods (such as BLASTp and ProteInfer), our EC-LMGraph yields just slightly (0.01) lower F1 score in terms of the 4th-digit prediction. This is primarily attributed to a fundamental disparity in training data scale. By utilizing massive sequence databases, sequence-based methods leverage a substantial volume of sequence examples to capture the fine-grained substrate specificities that characterize 4th-digit EC classes.
In contrast, EC-LMGraph is trained exclusively on proteins with experimentally validated PDB structures, as results in a substantially smaller annotated training dataset. To characterize this limitation explicitly, we have analyzed the class distribution of our training data, as now provided in Table S2. The analysis reveals that while the 1st-digit level contains abundant data, the dataset would fragment severely owing to the deepen functional hierarchy. At the 4th-digit level, the median drops to just 8 training samples per class, with 67.7% of all classes containing ≤ 10 examples.
Despite this constraint, EC-LMGraph remains highly competitive at the 4th-digit level, achieving an F1 of 0.54 compared to 0.55 and 0.54, respectively, for BLASTp and ProteInfer. This highlights that by incorporating a pre-trained protein language module, EC-LMGraph learns effectively from a substantially smaller structural dataset even under severe class sparsity at the 4th-digit level.
Table S2 and an expanded discussion of these challenges have been incorporated into the revised manuscript.
The aforementioned revisions are listed as follows:
Page 7, 3rd paragraph:
When breaking down the evaluation by independent hierarchical levels, EC-LMGraph consistently outperformed the other methods on broader functional classes, attaining the highest F1 scores for predicting the 1st-, 2nd-, and 3rd-digit EC classes (0.90, 0.85, and 0.82, respectively) (Fig. 2E). For the 4th-digit prediction, CLEAN-Contact achieved the highest F1 score (0.74), followed by DeepECTransformer (0.65). EC-LMGraph achieved results similar to those of BLASTp, CLEAN, and ProteInfer, with scores in the ~0.53–0.55 range (Fig. 2E). The relative performances remained consistent across sequence similarity levels (Fig. 2F). These results suggest that utilizing precise 3D structural graphs gives EC-LMGraph a distinct advantage in capturing the core catalytic mechanisms that define the 1st-, 2nd-, and 3rd-digit EC classes. In contrast, sequence-based methods utilize massive databases to access a substantial volume of sequence examples, effectively capturing the fine-grained substrate specificities that characterizing 4th-digit EC classes. An analysis of the EC-LMGraph training dataset reveals class imbalance as the functional hierarchy deepens (Table S2). While the 1st-digit level contains abundant data (median of 906 samples per class), the dataset would fragment severely owing to the deepen functional hierarchy. At the 4th-digit level, the median drops to just 8 training samples per class, with 67.7% of all classes containing ≤ 10 examples (Table S2). It is worth noting that by incorporating a protein language module pre-trained on large sequence databases, EC-LMGraph is able to learn through a substantially smaller annotated training

set (i.e., limited to proteins with experimentally validated structures), even when the numbers of samples in the 4th-digit classes are especially small.
Table S2:
Table S2: Distribution of training samples across the four hierarchical levels of the EC classification
EC Level | Description | Number of unique classes | Total training samples | Number of samples per class | Median samples per class | Classes with ≤ 10 samples | Classes with ≤ 50 samples
Level 1 | Main Class | (e.g., EC 1.-.-.-) | 7 | 12,230 | 1747.1 | 906 | 0 (0.0%) | 0 (0.0%)
Level 2 | Subclass | (e.g., EC 1.1.-.-) | 61 | 12,261 | 201 | 76 | 6 (9.8%) | 26 (42.6%)
Level 3 | Sub-subclass | (e.g., EC 1.1.1.-) | 175 | 11,886 | 67.9 | 28 | 41 (23.4%) | 117 (66.9%)
Level 4 | Substrate | (e.g., EC 1.1.1.1) | 561 | 7,497 | 13.4 | 8 | 380 (67.7%) | 541 (96.4%)

R1-6 Comment:
Comment 1:
6. In the section evaluating catalytic site prediction, the impact of enzyme length differences on the rationality of thresholds is not considered (e.g., the top 10% residues of short enzymes may far exceed the actual number of catalytic sites, while the top 10% residues of long enzymes may miss key sites).
Answer:
We sincerely thank the reviewer for this insightful comment. We intend to enrich our rationale of why the top 10% of predicted residues were counted in our consideration.
Catalytic residues typically comprise <1% of an enzyme's total sequence. Assessing the top 10% of predicted residues can simulate a practical experimental workflow for investigating unknown targets. In real-world de novo enzyme characterization, the absolute number of catalytic residues is fundamentally unknown. Establishing the top 10% of predicted residues as the putative active site could help prioritize the catalytic hotspots for ease of downstream experimental validation.
Moreover, the top 10% threshold is assumed not to introduce any length-dependent bias. To validate this assumption, we model a scenario with a total of K= 5 catalytic sites, then calculate the random probabilities for sequences with varying lengths (N = 100, 200, 400, and 1000). This process follows a hypergeometric distribution, as defined below:

Enzyme Length (N)	Search Space (n)	k = 1	k = 2	k = 3	k = 4	k = 5
100	10	33.94%	7.02%	0.64%	0.03%	< 0.01%
200	20	33.36%	7.16%	0.72%	0.03%	< 0.01%
400	40	33.08%	7.23%	0.77%	0.04%	< 0.01%
1000	100	32.92%	7.27%	0.79%	0.04%	< 0.01%
Limit ∞	10% of N	~32.80%	~7.29%	~0.81%	~0.05%	< 0.01%

This demonstrates that when the selection threshold is fixed at 10% of N, the probability of identifying k true sites remains nearly identical across all sequence lengths. Furthermore, in our analysis of the DeepFRI prediction results, we note that for some proteins, the model assigns very high activation values to a large majority (sometimes >80%) of the residues. Since the score distributions are highly skewed, applying a fixed, absolute probability threshold (such as 0.5) would make it impossible to determine an optimal cutoff that is fair to both algorithms. Furthermore, unlike GraphEC and other algorithms explicitly designed for catalytic site prediction, EC-LMGraph and DeepFRI do not incorporate any ground-truth catalytic site labels during its training. Since these models learn to localize sites inherently through functional predictions, their raw activation distribution naturally differs from models trained on residue-level labels. Therefore, thresholding raw probabilities would not lead to a fair comparison.
Comment 2:
Additionally, industry-common metrics such as the Precision-Recall (PR) curve or Matthews Correlation Coefficient (MCC) are not adopted, making it impossible to fully measure the balance between "prediction accuracy and coverage" and resulting in insufficient comprehensiveness of the evaluation results.
Answer:
We have now incorporated performance metrices including MCC score and Area Under the Precision-Recall Curve (PR-AUC), into the newly added Table S3. We observe that EC-LMGraph consistently outperforms DeepFRI across these benchmarks. Specifically, EC-LMGraph achieves substantially higher recall (0.390 vs. 0.224). It also achieves more than double the MCC score (0.109 vs. 0.047) and improves the PR-AUC by ~65% (0.220 vs. 0.133).
The aforementioned revisions are listed as follows:

Page 9, 2nd paragraph:
Since the exact number of catalytic sites in an uncharacterized enzyme is rarely known, establishing the top 10% of predicted residues as the putative active site could help prioritize the catalytic hotspots for ease of downstream experimental validation. This approach ensures standardized performance comparisons by evaluating an algorithm's capacity to rank actual catalytic residues within its top predictions Furthermore, this relative threshold does not introduce length-dependent bias, as the random probability of capturing true sites remains statistically constant regardless of enzyme length.
Page 10, 1st paragraph:
EC-LMGraph also consistently outperformed DeepFRI across all evaluation metrics (Table S3). Notably, it achieved substantially higher recall (0.390 vs. 0.224) for superior coverage, more than doubled the MCC score (0.109 vs. 0.047), and improved the area under the precision-recall curve by ~65% (0.220 vs. 0.133).
Table S3:
Table S3: performance comparison of catalytic site prediction between EC-LMGraph and DeepFRI

	EC-LMGraph	DeepFRI
Accuracy	0.896	0.892
Precision	0.050	0.029
Recall	0.390	0.224
Specificity	0.903	0.900
F1 Score	0.088	0.051
MCC	0.109	0.047
Macro PR-AUC	0.220	0.133

R1-7 Comment:
7. The impact of AlphaFold2 structural confidence on prediction need to be mentioned. In the section of proteome-wide enzyme prediction, AlphaFold2-predicted structures are used for analysis, yet the impact of structural confidence (pLDDT) is not considered. The differences in EC prediction F1 scores among the three structure groups (pLDDT > 90, 70 < pLDDT < 90, and pLDDT < 70) are not statistically analyzed. This makes it impossible to clarify the model's robustness to low-confidence predicted structures, thereby affecting the rigor of the results.
Answer:
Thank you for this suggestion. We agree that it is essential to assess the model's robustness relative to the structural confidence of the AlphaFold2-predicted structures. Therefore, we have conducted an additional analysis to investigate how structural confidence (pLDDT) would impact the prediction performance of EC-LMGraph. We have categorized the AlphaFold2-predicted structures into three groups based on their pLDDT scores: High confidence (pLDDT > 90), Medium confidence (70 < pLDDT ≤ 90), and Low confidence (pLDDT ≤ 70). Both the F1 scores and 95% confidence intervals (CI) across these groups (Fig. S6) are calculated. In the 3rd/4th-level EC predictions, our model achieves an F1 score of ~0.921 (95% CI: 0.920–0.922) for the high-confidence group and ~0.817 (95% CI: 0.814–0.820) for the medium-confidence group. Prediction accuracy decreases to ~0.615 (95% CI: 0.602–0.627) within the low-confidence group. This generally overviews how structural quality would influence the model's performance.
In the revised manuscript, we have included these statistical analyses and added Fig. S6 to illustrate the relationship between the pLDDT scores and the prediction performance.
The aforementioned revisions are listed as follows:
Page 11, 2nd paragraph:
As EC-LMGraph relies on the geometric arrangement of residues, the reliability of the AlphaFold2-predicted structure is crucial. We utilized the pLDDT (predicted Local Distance Difference Test), a per-residue confidence metric from AlphaFold2, to assess the confidence of the predicted atomic positions. To ensure the rigor of our predictions on computationally generated structures, we investigated the impact of these confidence scores on model performance by categorizing proteins into three groups: High (pLDDT > 90), Medium (70 < pLDDT ≤ 90), and Low (pLDDT ≤ 70). We observed that the quality of the predicted structure correlates with the accuracy of EC number prediction (Fig. S6). For the 3rd/4th-digit predictions, EC-LMGraph achieve an F1 scores of 0.921 (95% CI: 0.920–0.922) for high-confidence structures and 0.817 (95% CI: 0.814–0.820) for medium-confidence structures. Performance in the low-confidence group decreased to 0.615 (95% CI: 0.602–0.627). These results demonstrate that EC-LMGraph is highly effective for structures with high pLDDT scores, whereas predictions derived from low-confidence models (pLDDT ≤

70) should be interpreted with caution.

R1-8 Comment:
Comment 1:
8. In the discussion section, the paper mentions the latest methods such as ECPICK, DeepECTransformer, and GraphEC, but no direct quantitative comparison is conducted. Additionally, it does not compare the performance of EC-LMGraph (ProtT5 + GCN) and DeepECTransformer (ProtBert + CNN) under the "sequence-only input" scenario. As a result, the incremental value of GCN (structural information) in EC-LMGraph cannot be fully highlighted, leading to insufficient demonstration of its competitive advantages.
Answer:
Many thanks for your kind recommendation and comments. To address this, we have conducted new benchmarking experiments on a newly annotated sequence dataset to evaluate our framework against three additional models: DeepECTransformer, GraphEC, and ECPICK.
In the 3rd/4th-level EC evaluation (Fig. 2D), DeepECTransformer demonstrates strong performance with an F1 score of ~0.70, while EC-LMGraph achieves a highly competitive score of ~0.69. These two methods significantly outperformed ECPICK and GraphEC, which achieve F1 scores of only ~0.41 and ~0.38, respectively. When evaluating the hierarchical levels independently (Fig. 2E), our EC-LMGraph consistently outperforms the other methods on broader functional classes, attaining the highest F1 scores for predicting the 1st-, 2nd-, and 3rd-digit EC classes. These results suggest that utilizing precise 3D structural graphs can give EC-LMGraph a distinct advantage in capturing the core catalytic mechanisms that define these EC classes. For the 4th-digit prediction, DeepECTransformer achieves the highest F1 score of 0.65, followed by EC-LMGraph at 0.54. The remaining models, ECPICK and GraphEC, attained scores of 0.40 and 0.35, respectively. In our revised manuscript, we have updated the Results section to compare our presented EC-LMGraph against DeepECTransformer, GraphEC, and ECPICK (Fig. 2D, 2E, and 2F).
Comment 2:
It fails to compare the 3rd/4th-level EC F1 scores and catalytic site overlap rates between EC-LMGraph and GraphEC (which adopts ESMfold-predicted structures + graph model) on the same datasets (e.g., M-CSA, Parkinson’s disease-related proteins).
Regarding the comparison of catalytic site overlap rates between EC-LMGraph and GraphEC on M-CSA datasets, we found that a fair and direct quantitative comparison is unfortunately infeasible. The GraphEC framework incorporates catalytic site labels directly into its training phase. Through a cross-check of the datasets, we found that the catalytic site annotations of over 200 M-CSA structures were already included in GraphEC’s training set. Consequently, evaluating GraphEC on these M-CSA targets would result in severe data leakage.
In contrast, EC-LMGraph and DeepFRI do not utilize active site labels during training. Instead, they rely on explainable AI methods, such as saliency maps or Grad-CAM, to pinpoint physical active sites without prior knowledge. Therefore, a direct quantitative comparison between the supervised catalytic site predictions (i.e. GraphEC) and the unsupervised approaches (i.e. EC-LMGraph, DeepFRI) would result in a biased comparison.
The aforementioned revisions are listed as follows:
Page 7, 3rd paragraph:
We then compared the performance of EC-LMGraph against eleven other methods, including the sequence alignment method (BLASTp) [3], sequence-based deep learning methods (CLEAN [4], CLEAN-Contact [5], DeepECTransformer [6], ProteInfer [7], ECPred [8], DeepEC [9], GraphEC [10], and ECPICK [11]), and structure-based methods (DeepFRI [16] and COFACTOR [15]). In the overall combined 3rd/4th-level EC evaluation, CLEAN-Contact and DeepECTransformer showed strong performance with F1 scores of ~0.78 and ~0.70, respectively, while EC-LMGraph followed closely behind (~0.69) (Fig. 2D). When breaking down the evaluation by independent hierarchical levels, EC-LMGraph consistently outperformed the other methods on broader functional classes, attaining the highest F1 scores for predicting the 1st-, 2nd-, and 3rd-digit EC classes (0.90, 0.85, and 0.82, respectively) (Fig. 2E). For the 4th-digit prediction, CLEAN-Contact achieved the highest F1 score (0.74), followed by DeepECTransformer (0.65). EC-LMGraph achieved results similar to those of BLASTp, CLEAN, and ProteInfer, with scores in the ~0.53–0.55 range (Fig. 2E). The relative performances remained consistent across sequence similarity levels (Fig. 2F). These results suggest that utilizing precise 3D structural graphs gives EC-LMGraph a distinct advantage in capturing the core catalytic mechanisms that define the 1st-, 2nd-, and 3rd-digit EC classes. In contrast, sequence-based methods utilize massive databases to access a substantial volume of sequence examples,

effectively capturing the fine-grained substrate specificities characterizing 4th-digit EC classes.

Reviewer 2:
Comment:
The manuscript by Ng et al. presents a protein language model- and graph convolutional network-based framework to predict enzyme commission (EC) numbers from protein sequence features and structures, and saliency mapping to score representative residues attributing to the enzymatic functions. The introduction provides a clear overview of the research gap and places the methodology in context. Methodology is clearly described and evaluated in a proper way. Model interpretation to predict catalytic sites is presented as well.
Answer:
Many thanks for your kind recommendation and comments, to which we reply point-by-point as follows, along with the modifications in our new version of manuscript.

R2-1 Comment:
1) How about influence of environmental/experimental conditions?
Answer:
We sincerely thank the reviewer for this insightful comment. We agree that environmental and experimental conditions are critical factors in enzymatic function. However, standardized datasets linking 3D structures with precise environmental metadata do remain scarce. This data limitation has prevented the inclusion of these variables in the current version of EC-LMGraph. Future advancements in high-throughput assays and molecular dynamics simulations will be vital to addressing this gap. We have added a paragraph to the Discussion section to explain these data bottlenecks and also to outline potential strategies for future integration.
The aforementioned revisions are listed as follows:
Page 13, 3rd paragraph:
EC-LMGraph currently utilizes static structural representations that do not explicitly account for environmental conditions like pH or temperature. While an enzyme's intrinsic 3D fold dictates its fundamental EC classification, environmental factors largely influence its reaction kinetics and conformational dynamics. Incorporating environmental metadata is currently bottlenecked by data scarcity, future integration of high-throughput assays or molecular dynamics simulations could potentially overcome these hurdles.

R2-2 Comment:
2) How about multi-label cases (enzymes involved in multiple different reactions)?
Answer:
Many thanks for your kind recommendation. We have designed EC-LMGraph as a multi-label framework that calculates independent probabilities for every EC category. The final layer of the neural network comprises a number of neurons equal to the total count of EC classes, with each output neuron activated by an independent sigmoid function. As these functions operate independently, the model can simultaneously assign high probabilities to multiple classes. Therefore, if an enzyme structure matches the characteristics of multiple functional classes, the model is capable of generating all relevant EC labels as the model output.
We have updated the manuscript to introduce how our framework does handle multi-functional cases, followed by the discussion about the focal loss implementation.
The aforementioned revisions are listed as follows:
Page 5, 2nd paragraph:
Many enzymes in nature are multi-functional, catalyzing multiple distinct reactions and thus carrying several EC numbers. To accommodate these cases, we have designed EC-LMGraph to compute independent probabilities for every EC class across the functional hierarchy (See Methods).
Page 18, 1st paragraph:
At last, a fully connected layer is employed, where the number of output neurons corresponds to the total number of EC classes. To support multi-label prediction, each output neuron is activated by an independent sigmoid function, i.e., . This activation function maps the output of neurons to a value between 0 and 1,

representing the probability belonging to the specific EC class. These independent sigmoid functions allow the model to simultaneously assign high probabilities to multiple classes.

R2-3 Comment:
3) Proper attention is given to existing related work in terms of enzyme function prediction. However, recently approaches have also been suggested for prediction of enzymatic parameters. It could be useful to briefly comment on such methodology as well in order to further expand the description of the context of this work.
Answer:
We thank the reviewer for highlighting the importance of enzymatic parameter prediction. To broaden the study's context, we have added a paragraph to discuss the kinetic parameter prediction models, including UniKP and DEKP, in the revised manuscript. As accurate EC classification is a prerequisite for kinetic parameter estimation, our EC-LMGraph provides the essential functional foundation to support these downstream tasks and potentially to enable end-to-end frameworks.
The aforementioned revisions are listed as follows:
Page 13, 3rd paragraph:
In addition to function predictions, recent deep learning approaches have also advanced in predicting enzymatic kinetic parameters. Methodologies utilizing protein language models and graph neural networks, such as UniKP [64] and DEKP [65], have been successfully applied to estimate turnover numbers and Michaelis constants directly from protein sequences and substrate structures. While these tools focus on the quantitative mechanics of enzyme-substrate efficiency, EC-LMGraph could complement this broader ecosystem by focusing on the preceding step: accurate, structure-aware prediction of the enzyme function and the localization of its catalytic sites.
References
64. Yu H, Deng H, He J, Keasling J D, Luo X. UniKP: a unified framework for the prediction of enzyme kinetic parameters. Nat Commun. Springer US; 2023; doi: 10.1038/s41467-023-44113-1.
65. Wang Y, Cheng L, Zhang Y, Cao Y, Alghazzawi D. DEKP: A deep learning model for enzyme kinetic parameter prediction based on pretrained models and graph neural networks. Brief Bioinform. 2025; doi: 10.1093/bib/bbaf187.

R2-4 Comment:
4) It is not fully clear to me if all relevant data and code are available. In particular, can I download and access (i) the trained model; (ii) the code used to train the model (not just the code to run prediction); (iii) predicted enzymatic functions which are described at large scale; (iv) predicted catalytic sites.
Answer:
Many thanks for your comments. To address your concerns, we have updated our GitHub repository (https://github.com/ngyuilun/EC-LMGraph). Users can now find the trained model, as well as the code used to train the model, in the repository. Furthermore, we have also uploaded large-scale prediction results regarding the enzymatic functions and their catalytic sites.

R2-5 Comment:
5) Minor spelling issue: "The prediction performance of EC-LMGraph certainly still has rooms for further improvement": rooms--> room.
Answer:
Many thanks for pointing out this grammatical error. We have corrected the typo in the revised manuscript.
Page 13, 3rd paragraph:
The prediction performance of EC-LMGraph certainly still has room for further improvement.

Cookie Preference Center

We use cookies which are necessary to make our site work. We may also use additional cookies to analyze, improve and personalize our content and your digital experience. For more information, see our Cookie Policy.

You may choose not to allow some types of cookies. However, blocking some types may impact your experience of our site and the services we are able to offer. See the different category headings below to find out more or change your settings.

Allow all

Manage Consent Preferences

Strictly Necessary Cookies

Always active

These cookies are necessary for the website to function and cannot be switched off in our systems. They are usually only set in response to actions made by you which amount to a request for services, such as setting your privacy preferences, logging in or filling in forms. You can set your browser to block or alert you about these cookies, but some parts of the site will not then work.

Functional Cookies

These cookies enable the website to provide enhanced functionality and personalisation. They may be set by us or by third party providers whose services we have added to our pages. If you do not allow these cookies then some or all of these services may not function properly.

Performance Cookies

These cookies allow us to count visits and traffic sources so we can measure and improve the performance of our site. They help us to know which pages are the most and least popular and see how visitors move around the site.

Targeting Cookies

These cookies may be set through our site by our advertising partners. They may be used by those companies to build a profile of your interests and show you relevant adverts on other sites. If you do not allow these cookies, you will experience less targeted advertising.

Cookie List

Clear

Apply Cancel

Consent Leg.Interest

Confirm my choices

Power
