# Supplementary material for: Accurate proteome-wide prediction of enzymes and catalytic sites using graph deep learning and protein language model
Source: Gigascience. 2026 May 13;15:giag056. doi: 10.1093/gigascience/giag056 (PMC13217607; doi:10.1093/gigascience/giag056)
Supplement: giag056_Reviewer_1_Report_original_submission — Reviewer 1 -- 9/20/2025 [file giag056_reviewer_1_report_original_submission.pdf]

Reviewer Report

Title: Accurate proteome-wide prediction of enzymes and catalytic sites using graph deep learning and protein language model

Version: Original Submission **Date:** 9/20/2025

Reviewer name: Reviewer 1

Reviewer Comments to Author:

Gerneal Comments
The EC-LMGraph framework proposed in this paper innovatively combines protein language models with graph convolutional networks, realizing the collaborative optimization of 3rd/4th-level EC number prediction for enzymes and catalytic site localization. Meanwhile, it expands the application scenarios of the whole proteome through AlphaFold2-predicted structures. The study features a rigorous design, comprehensive verification dimensions, and a good balance between interpretability and computational efficiency. Compared with existing methods, it shows obvious advantages and holds significant reference value for enzyme function annotation and experimental design.
However, the paper still has room for improvement in terms of the completeness of methodological details, the depth of result analysis, and the comparison with the latest methods. Further experimental evidence and logical explanations need to be supplemented to enhance the persuasiveness of the conclusions.
1. The paper states that "9Ã… is the optimal distance threshold", yet only mentions the optimization range (6-13Ã…) in Supplementary Figure S1B. The following information is not clearly specified in the main text: The specific impact of different thresholds (e.g., 6Ã…, 9Ã…, 12Ã…) on the prediction performance of 3rd/4th-level EC numbers (such as differences in F1 score, precision, and recall), particularly whether there exists a threshold preference when predicting different major EC classes (e.g., EC 3 hydrolases, EC 7 translocases). Overall the author should comment on insufficient rationale for distance threshold optimization in protein graph construction; The quantitative relationship between thresholds and computational efficiency (e.g., a 1Ã… increase results in an 11.3% rise in computation time, but is the corresponding performance improvement significant?).
2. The author directly adopts ProtT5-XL-U50 as the sequence feature extraction module, yet fails to explain why other mainstream pLMs are not chosen. It is advisable to add comparative experiments of pLMs (including at least 1-2 mainstream models) or clarify the core basis for selecting ProtT5 in the discussion section.
3. The EC prediction need to compare with CLEAN-Contact, the newly published paper.
4. Lack of Basis for Parameter Setting of Focal Loss. In the paper, the focusing parameter Î³ is fixed at 2, yet no explanation is provided for why this value is chosen. It is recommended to supplement the comparison of F1 scores under different Î³ values and clarify how Î³=2 optimally addresses the sample imbalance issue in 4th-level EC prediction.
5. The paper mentions that "on AlphaFold2-predicted structures, the F1 score for 4th-level EC prediction is 0.54, which is lower than that of BLASTp (0.55) and ProteInfer (0.54)", yet it fails to conduct an in-

depth analysis of the reasons behind this phenomenon.

6. In the section evaluating catalytic site prediction, the impact of enzyme length differences on the rationality of thresholds is not considered (e.g., the top 10% residues of short enzymes may far exceed the actual number of catalytic sites, while the top 10% residues of long enzymes may miss key sites). Additionally, industry-common metrics such as the Precision-Recall (PR) curve or Matthews Correlation Coefficient (MCC) are not adopted, making it impossible to fully measure the balance between "prediction accuracy and coverage" and resulting in insufficient comprehensiveness of the evaluation results.

7. The impact of AlphaFold2 structural confidence on prediction need to be mentioned. In the section of proteome-wide enzyme prediction, AlphaFold2-predicted structures are used for analysis, yet the impact of structural confidence (pLDDT) is not considered. The differences in EC prediction F1 scores among the three structure groups (pLDDT > 90, 70 < pLDDT < 90, and pLDDT < 70) are not statistically analyzed. This makes it impossible to clarify the model's robustness to low-confidence predicted structures, thereby affecting the rigor of the results.

8. In the discussion section, the paper mentions the latest methods such as ECPICK, DeepECTransformer, and GraphEC, but no direct quantitative comparison is conducted. It fails to compare the 3rd/4th-level EC F1 scores and catalytic site overlap rates between EC-LMGraph and GraphEC (which adopts ESMfold-predicted structures + graph model) on the same datasets (e.g., M-CSA, Parkinson's disease-related proteins). Additionally, it does not compare the performance of EC-LMGraph (ProtT5 + GCN) and DeepECTransformer (ProtBert + CNN) under the "sequence-only input" scenario. As a result, the incremental value of GCN (structural information) in EC-LMGraph cannot be fully highlighted, leading to insufficient demonstration of its competitive advantages.

Methods

Are the methods appropriate to the aims of the study, are they well described, and are necessary controls included? Choose an item.

Conclusions

Are the conclusions adequately supported by the data shown? Choose an item.

Reporting Standards

Does the manuscript adhere to the journal's guidelines on minimum standards of reporting? Choose an item.

Choose an item.

Statistics

Are you able to assess all statistics in the manuscript, including the appropriateness of statistical tests used? Choose an item.

Quality of Written English

Please indicate the quality of language in the manuscript: Choose an item.

Declaration of Competing Interests

Please complete a declaration of competing interests, considering the following questions:

- Have you in the past five years received reimbursements, fees, funding, or salary from an organisation that may in any way gain or lose financially from the publication of this manuscript, either now or in the future?
- Do you hold any stocks or shares in an organisation that may in any way gain or lose financially from the publication of this manuscript, either now or in the future?
- Do you hold or are you currently applying for any patents relating to the content of the manuscript?
- Have you received reimbursements, fees, funding, or salary from an organization that holds or has applied for patents relating to the content of the manuscript?
- Do you have any other financial competing interests?
- Do you have any non-financial competing interests in relation to this paper?

If you can answer no to all of the above, write 'I declare that I have no competing interests' below. If your reply is yes to any, please give details below.

I declare that I have no competing interests

I agree to the open peer review policy of the journal. I understand that my name will be included on my report to the authors and, if the manuscript is accepted for publication, my named report including any attachments I upload will be posted on the website along with the authors' responses. I agree for my report to be made available under an Open Access Creative Commons CC-BY license (http://creativecommons.org/licenses/by/4.0/). I understand that any comments which I do not wish to be included in my named report can be included as confidential comments to the editors, which will not be published.

Choose an item.

To further support our reviewers, we have joined with Publons, where you can gain additional credit to further highlight your hard work (see: https://publons.com/journal/530/gigascience). On publication of this paper, your review will be automatically added to Publons, you can then choose whether or not to claim your Publons credit. I understand this statement.

Yes Choose an item.
